# Spatio-temporal coupled mode theory for nonlocal metasurfaces

**DOI:** 10.1038/s41377-023-01350-9

**Published:** 2024-01-24

**Authors:** Adam Overvig, Sander A. Mann, Andrea Alù

**Affiliations:** 1 Photonics Initiative, Advanced Science Research Center, City University of New York, New York, NY 10031 USA; 2 Physics Program, Graduate Center of the City University of New York, New York, NY 10016 USA

**Keywords:** Nanophotonics and plasmonics, Slow light

## Abstract

Diffractive nonlocal metasurfaces have recently opened a broad range of exciting developments in nanophotonics research and applications, leveraging spatially extended—yet locally patterned—resonant modes to control light with new degrees of freedom. While conventional grating responses are elegantly captured by temporal coupled mode theory, current approaches are not well equipped to capture the arbitrary spatial response observed in the nascent field of nonlocal metasurfaces. Here, we introduce spatio-temporal coupled mode theory (STCMT), capable of elegantly capturing the key features of the resonant response of wavefront-shaping nonlocal metasurfaces. This framework can quantitatively guide nonlocal metasurface design while maintaining compatibility with local metasurface frameworks, making it a powerful tool to rationally design and optimize a broad class of ultrathin optical components. We validate this STCMT framework against full-wave simulations of various nonlocal metasurfaces, demonstrating that this tool offers a powerful semi-analytical framework to understand and model the physics and functionality of these devices, without the need for computationally intense full-wave simulations. We also discuss how this model may shed physical insights into nonlocal phenomena in photonics and the functionality of the resulting devices. As a relevant example, we showcase STCMT’s flexibility by applying it to study and rapidly prototype nonlocal metasurfaces that spatially shape thermal emission.

## Abstract (web summary)

Nonlocal metasurfaces with spatially varying geometries are modeled using a generalized coupled mode theory that operates in real space, enabling rapid numerical prototyping and insightful modeling of their spectro-spatial features.

## Graphical abstract

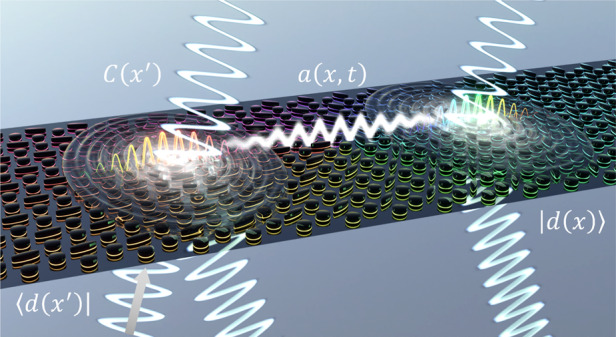

## Introduction

Coupled mode theory (CMT) has become a powerful tool to model and understand the scattering response of complex resonant electromagnetic structures^1^. Central to CMT is the phenomenological identification of (i) the resonant modes supported by the system, and (ii) ports, or channels, that carry energy to and from the resonant scatterer. CMT provides an intuitive and accurate framework to study a wide range of resonant devices. In the spatial domain^2–4^, it is commonly used to study waveguide modes and their coupling; while in the time domain temporal coupled mode theory (TCMT)^5,6^ has found marked success in modeling Fano resonances and in the construction of scattering matrices. This technique can be applied both to localized resonances^7–9^ and to extended resonances supported by metasurfaces, photonic crystal slabs and subwavelength gratings^10,11^. However, in TCMT the spatial distribution of the mode is ignored: the modal amplitude $a$ is assumed to be function only of time. This feature limits TCMT to studying the spectral features of the device, while detailed spectro-spatial information of the modes themselves is not captured. For this reason, TCMT is primarily effective at studying infinitely periodic grating structures, while finite and spatially varying gratings are not straightforwardly modeled.

**Fig. 1:**
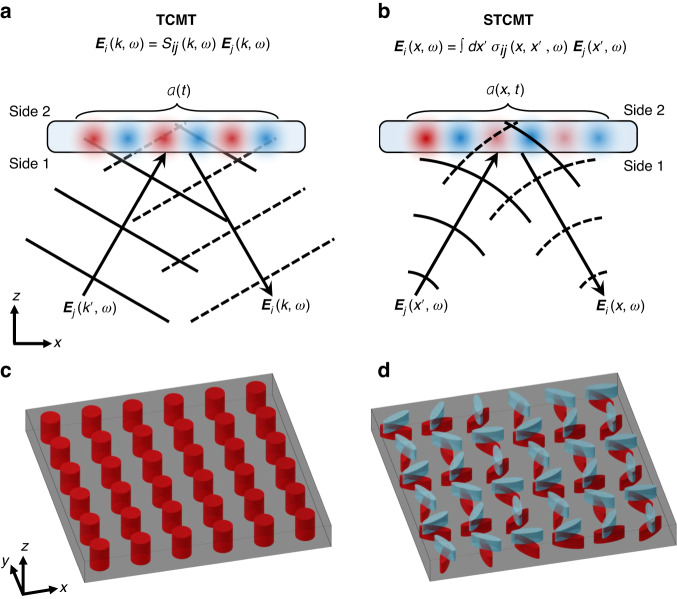
Coupled mode theories. **a** TCMT is effective at modeling systems in which the complex modal amplitude *a*(*t*) is spatially invariant, wherein the scattered field ${{\bf{E}}}_{j}$ is the product of the scattering matrix ${S}_{ij}$ and the incident field ${{\bf{E}}}_{i}$. **b** STCMT is necessary when the complex modal amplitude $a(x,t)$ is spatially varying, wherein the scattered field ${{\bf{E}}}_{j}$ is the convolution of the scattering kernel ${\sigma }_{ij}$ and the incident field ${{\bf{E}}}_{i}$. **c** Example conventional nonlocal metasurface with spatially invariant meta-unit cells. **d** Example diffractive nonlocal metasurface with spatially varying meta-unit cells, varying from left to right

Yet, the spectro-spatial properties of photonic resonant systems are important in various settings, particularly when the optical energy is correlated across distances larger than the wavelength—i.e., when nonlocality, also known as spatial dispersion, cannot be neglected. In turn, these systems exhibit strong dependence of the optical response on the momentum of light^12,13^. In dielectric thin film platforms, the dispersion of guided modes provides an engineerable mechanism to control nonlocal phenomena^14–16^. In this context, nonlocal metasurfaces^17–19^ have been garnering attention for their capability of enhancing the control of light by tailoring it in momentum space, of particular interest for signal processing^18^, image differentiation^19,20^, spectral^21^ and chiral sensing^22^, augmented reality applications^23^ and for compactifying free space^24,25^. This class of devices employs subwavelength, transversely invariant structures, hence the spatial features of their extended resonant modes do not escape the near field of the device. As a result, TCMT remains effective, lumping the resonance into a spatially invariant modal parameter $a(t)$ [Fig. 1a].

Recent reviews^26^, perspectives^27–29^ and studies on the fundamental role of nonlocality in optics^30^ suggest that embracing nonlocality is a budding area of inquiry and a compelling direction for the maturing field of metasurfaces. In particular, engineered nonlocalities can be combined with locally varying features to enable *diffractive nonlocal metasurfaces*^28^. These ultrathin optical devices support highly selective resonant responses combined with tailored spatial variations, which are capable of patterning the optical wavefront engaging the resonance^31–39^. Leveraging the high-Q physics of quasi-bound states in the continuum (q-BICs)^40^, these devices support long-lived, spatially extended resonant states that couple to free-space with amplitude, phase and polarization properties spatially tailored across the aperture through symmetry-controlled perturbations. By spatially varying the perturbations with a uniform phase gradient, for instance, light can be anomalously diffracted with near-unity efficiency and circular polarization selectivity^33^. By nonperiodically tailoring the local perturbations across the device, the resonant response can efficiently engage arbitrarily tailored optical wavefronts of choice. As a remarkable example, a nonlocal metalens with hyperbolic phase distribution across the aperture can selectively engage only fields originating from its focal spot, while it remains transparent for other wavefront shapes^34^. However, since their response is spectro-spatial in a nontrivial way, it cannot be captured by TCMT. Rather, a new theoretical framework featuring a spatially varying modal parameter $a(x,t)$ is required [Fig. 1b]. Figure 1c depicts a conventional nonlocal metasurface whose meta-unit cells are subwavelength and transversely invariant, hence compatible with a TCMT description. Figure 1d on the contrary depicts an example diffractive nonlocal metasurface (based on ref. ^33^) with a spatially varying orientation angle, incompatible with a TCMT description and requiring a generalized CMT model.

Here, we introduce a spatio-temporal coupled mode theory (STCMT) that extends TCMT to capture spatially varying resonant modes. We show excellent agreement with full-wave simulations of spatially selective diffractive nonlocal metasurfaces^34^, demonstrating the validity and relevance of this model. Our theory introduces a *nonlocality length*, which quantifies the degree of nonlocality of a given device, and it establishes how this parameter directly controls the degree of spatial selectivity. We clarify two distinct regimes of operation for nonlocal photonic devices, governed by the numerical aperture of the anomalous diffraction encoded into the device, and we demonstrate the relation between spatial selectivity and the supported eigenmodes. While recent efforts on spatio-temporal coupled mode theory have focused on temporally varying systems^41^ or conventional low-contrast grating systems^42^, our work provides a broad and much-needed framework for the rapidly emerging field of spatially varying and finite nonlocal metasurfaces. Our STCMT elegantly captures the fundamental working mechanisms of spatially varying resonant photonic systems, provides physical insights, and enables rational designs and rapid, computationally efficient analysis of next-generation photonic systems.

## Results

In the following, we develop STCMT by writing down the appropriate dynamical equations, determining the constraints stemming from time-reversal invariance, reciprocity and energy conservation, and then computing the scattering from a general nonlocal metasurface by appropriately specifying its parameters.

We first describe the nature of the problem. Coupled mode theories are useful because they eliminate the full vectoral information of Maxwell’s equations in favor of a few modal parameters and scattering coefficients involving a finite number of input and output ports. In STCMT, we seek to assign the spatial and temporal properties of the scattering into a single modal parameter $a(x,t)$. For simplicity, here we develop STCMT for a single mode; our approach may be generalized for multiple modes. While STCMT applies more broadly, it is instructive to consider a concrete system. As our archetypical example: a bound state in the continuum (BIC) is a bound mode in a nonlocal metasurface with a complex modal field ${E}_{BIC}(x,y,z)$ that can be treated as invariant to time (other than the harmonic response $\exp (-i\omega t)$); it is an eigenstate of a closed system. This closed system is typically infinitely periodic with subwavelength periodicity—the mode profile has subwavelength spatial details (“microstructure”) that repeat indefinitely without change across the aperture of the device (it has no “macrostructure”). To access it from free space, it may be perturbed into a q-BIC with a modal field ${E}_{qBIC}(x,y,z,t)$, which is a quasi-normal mode of an open system that leaks to the radiation continuum and therefore will generally vary with time (e.g., decay away). In a weakly perturbed system, we approximate this field as^34^

1 $${E}_{qBIC}(x,y,z,t)\approx a(t){E}_{BIC}(x,y,z)$$

where the spatial part of the q-BIC is treated as identical to that of the BIC, while the $a(t)$ captures the time dependence introduced by the perturbation. In TCMT, after assuming that the characteristic time scale on which $a(t)$ varies is much slower than the resonant frequency ${\omega }_{res}$, we may solve the scattering problem solely in terms of this modal parameter $a(t)$, or its Fourier counterpart $a(\omega )$. In other words, we simplify the scattering problem into solely solving for the “temporal macrostructure” of the system, which is sufficient to capture the spectral features of the q-BIC (i.e., Fano resonant line shapes).

Here, the coupled mode theory we seek will additionally capture the spatial properties of a mode in a *spatially varying system* in a parallel way. In such a system, the perturbation transforming the BIC to a q-BIC varies across the device and so Eq. (1) becomes

2 $${E}_{qBIC}(x,y,z,t)\approx a(x,t){E}_{BIC}(x,y,z)$$

where we now include the spatial macrostructure of the mode into $a(x,t)$. In close analogy to the temporal macrostructure in TCMT, in STCMT, we assume this macrostructure varies slowly compared to the microstructure, i.e., at length scales larger than the unit cell of a nonlocal metasurface. In this way, we may retain the interesting aspects of the spatially varying mode, while still ignoring the detailed microstructure of the modes. STCMT therefore naturally extends the phenomenology of TCMT to the spatial domain, capturing the *spectrospatial* behavior of finite and spatially varying nonlocal metasurfaces.

### Dynamical equations

We obtain the spatio-temporal dynamical equations by appropriately manipulating and tailoring standard TCMT equations^5^. Under an ${e}^{-i\omega t}$ time convention, and for a single resonance, these equations are

3 $$\frac{{\rm{d}}a(t)}{{\rm{d}}t}=-i\varOmega a(t)+\langle {\kappa }^{\ast }|{s}_{+}\rangle$$

4 $$|{s}_{-}(t)\rangle =C|{s}_{+}(t)\rangle +a(t)|d\rangle$$

Equation (3) describes the dynamics of the complex modal amplitude $a(t)$ excited by an incoming wave $|{s}_{+}(t)\rangle$, where ${|a(t)|}^{2}$ is normalized as the energy stored in the q-BIC per unit area at time $t$ and $\langle {s}_{+}|{s}_{+}\rangle$ is the incident intensity. The in-coupling vector $|\kappa \rangle$ contains the coupling coefficients to the mode from free-space excitation, and $\varOmega ={\omega }_{0}-i\gamma$ is a complex frequency, with ${\omega }_{0}$ being the (resonant) modal frequency and $\gamma$ being the total scattering rate. Equation (4) describes the dynamics of the outgoing (scattered) waves $|{s}_{-}\rangle$ (also normalized such that $\langle {s}_{-}|{s}_{-}\rangle$ is the outgoing intensity) as the interference of the “background” scattering, described by the matrix $C$, and the mode leaking to free space, described by the out-coupling vector $|d\rangle$ (see Supplementary Section S1 for additional background).

In contrast, the dynamical equations for a device with space-varying modal properties must inherently depend on the nature of the mode itself. In other words, we need at the outset to know the form of the dispersion of the mode. We expect, for instance, the phenomenology of a traveling wave to differ from that of a standing wave, and hence we expect distinct dynamical equations. A general approach to capture this dependence is a Taylor expansion for the resonant frequency in terms of momentum $k$:

5 $$\varOmega (k)=\left({\omega }_{0}+ck+\frac{b}{2}{k}^{2}+\mathrm{..}.\right)-i\left({\gamma }_{0}+{\gamma }_{1}k+\frac{{\gamma }_{2}}{2}{k}^{2}\mathrm{..}.\right)$$

where $k$ is the transverse momentum, ${\omega }_{0},c,b$ are the coefficients for the real part of the resonant frequency, and ${\gamma }_{0},{\gamma }_{1},{\gamma }_{2}$ are the coefficients for the imaginary part of the resonant frequency. For instance, a traveling wave is captured by the linear term with speed $c$, while a standing wave with parabolic dispersion, such as near a band edge, is captured by the coefficient $b$. As detailed in Supplementary Section S2, transforming Eqs. (3) and (4) into the frequency domain, including its momentum dependence and the expansion (5), and then transforming to the space-time domain, yields the dynamical equations (up to second order)

6 $$\begin{array}{l}\frac{{\rm{d}}a(x,t)}{{\rm{d}}t}+i({\omega }_{0}-i{\gamma }_{0})a(x,t)+i(c-i{\gamma }_{1})\frac{{\rm{d}}a(x,t)}{{\rm{d}}x}\\\qquad\quad+\,i(b-i{\gamma }_{2})\frac{{{\rm{d}}}^{2}a(x,t)}{{\rm{d}}{x}^{2}}=\int {\rm{d}}x^{\prime} \langle \kappa^{\ast } (x,x^{\prime} )|{s}_{+}(x^{\prime} )\rangle\end{array}$$

7 $$|{s}_{-}(x)\rangle =\int {\rm{d}}x^{\prime} (C(x,x^{\prime} )|{s}_{+}(x^{\prime} )\rangle +a(x)|d(x,x^{\prime} )\rangle )$$

Equations (6) and (7) are the dynamical equations for STCMT, including spatial dispersion in both the *coupling* properties (e.g., convolution terms on the right-hand sides) and the *modal* properties (spatial derivative terms on the left-hand sides). We note that this is can be readily extended to higher order expansions if needed, but in the most common scenarios the coefficients in Eq. (6) are more than sufficient. For instance, a pure traveling wave is characterized by a first-order spatial derivative, with Eq. (6) simplifying to a form akin to the one-way wave equation (see ref. ^42^); a standing wave at a band edge, modeled parabolically, is characterized by a second-order spatial derivative, with Eq. (6) simplifying to a Schrödinger equation (as we explore below). The coefficients $c,b$ therefore quantify the spatial dispersion of two modes of different type, each of which can be captured by STCMT. Similarly, the radiative decay profile, captured by the coefficient ${\gamma }_{1,2}$, also describes a nonlocal effect.

Next, we specialize our general STCMT to capture the functionalities demonstrated by recent diffractive nonlocal metasurfaces^31–37^. These devices leverage q-BIC modes, whose Q-factors are sufficiently large such that the background varies slowly compared to the resonant features^5^. In their infinitely periodic implementation, they abide a TCMT description with $c={\gamma }_{1}={\gamma }_{2}=0$ near normal incidence: they are well captured by a parabolic band with a Q-factor and resonant polarization state that is not a function of the incident angle. Their design assumes that the scattering of a q-BIC can be spatially tailored by locally changing a small symmetry-breaking perturbation, and that distant perturbations do not affect the local polarization or phase^31^. Hence, the nonlocality of these devices is assumed to be purely *modal*, or captured by the spatial derivatives in the left-hand side, while the *coupling* to and from the mode is spatially instantaneous (local):

8 $$\begin{array}{c}C(x,x^{\prime})\propto \delta (x-x^{\prime} )\\ |\kappa (x,x^{\prime})\rangle \propto \delta (x-x^{\prime} )\\ |d(x,x^{\prime})\rangle \propto \delta (x-x^{\prime} )\end{array}$$

Together, these assumptions yield the dynamical equations

9 $$\frac{{\rm{d}}a(x,t)}{{\rm{d}}t}+i({\omega }_{0}-i\gamma )a(x,t)+ib\frac{{{\rm{d}}}^{2}a(x,t)}{{\rm{d}}{x}^{2}}=\langle \kappa^{\ast } (x)|{s}_{+}(x,t)\rangle$$

10 $$|{s}_{-}(x,t)\rangle =C(x)|{s}_{+}(x,t)\rangle +a(x,t)|d(x)\rangle$$

where we simplify the notation to $\gamma ={\gamma }_{0}$. Supplementary Section S2b derives the same set of equations using the leading order terms of a cosine series expansion.

### Scattering Kernel and Green’s function

In TCMT, the scattering matrix is obtained by assuming time-harmonic solutions, solving Eq. (3) for $a(t)$, and then inserting the result into Eq. (4) to yield^5^

11 $$|{s}_{-}(\omega )\rangle =S(\omega )|{s}_{+}(\omega )\rangle$$

12 $$S(\omega )=C(\omega )+\frac{|d\rangle \langle {\kappa}^{\ast }|}{i(\varOmega -\omega )}$$

In comparison, in STCMT we must solve Eq. (9) before inserting it into Eq. (10). To do so, we recognize that, when no source is present, we obtain a Schrödinger equation with complex potential $V={\omega }_{0}-i\gamma$, recognizable by the rearrangement:

13 $$i\frac{{\rm{d}}a(x,t)}{{\rm{d}}t}=-\frac{b}{2}\frac{{{\rm{d}}}^{2}a(x,t)}{{\rm{d}}{x}^{2}}+Va(x,t)$$

Compared to the conventional Schrödinger equation, we have $\hslash =1$ and $b=1/m$, consistent with the expectation that the band curvature plays the role of an inverse effective mass. We also note that non-Hermiticity (a complex valued potential) is expected of an open system. This well-studied equation abides solutions via the Propagator, or Green’s function (Supplementary Section S3).

In practice, we are most often interested in the response to monochromatic waves. In this case, Eq. (9) becomes

14 $$i({\omega }_{0}-\omega -i\gamma )a(x,\omega )+ib\frac{{{\rm{d}}}^{2}a(x,\omega )}{{\rm{d}}{x}^{2}}=\langle \kappa^{\ast } (x)|{s}_{+}(x,\omega )\rangle$$

and we seek Green’s function satisfying

15 $$i({\omega }_{0}-\omega -i\gamma )G(x,x^{\prime} ,\omega )+ib\frac{{{\rm{d}}}^{2}G(x,x^{\prime} ,\omega )}{{\rm{d}}{x}^{2}}=\delta (x-x^{\prime} )$$

which yields the modal amplitude

16 $$a(x,\omega )=\int {\rm{d}}x^{\prime} G(x,x^{\prime} ,\omega )\langle \kappa^{\ast } (x^{\prime} )|{s}_{+}(x^{\prime} ,\omega )\rangle$$

Insertion of Eq. (16) into the space-frequency form of Eq. (10) yields the scattering equation

17 $$|{s}_{-}(x,\omega )\rangle =\int {\rm{d}}x^{\prime} \sigma (x,x^{\prime} ,\omega )|{s}_{+}(x^{\prime} ,\omega )\rangle$$

18 $$\sigma (x,x^{\prime} ,\omega )=C(x,\omega )\delta (x-x^{\prime} )+G(x,x^{\prime} ,\omega )|d(x)\rangle \langle {\kappa }^{\ast }(x^{\prime} )|$$

where the scattering kernel $\sigma$ correlates input positions $x^{\prime}$ with output positions $x$, and where the closed form of Green’s function is

19 $$G(x,x^{\prime} ,\omega )=\frac{i}{b}\xi (\omega )\exp \left(-\frac{|x-x^{\prime} |}{\xi (\omega )}\right)$$

where

20 $$\xi (\omega )=\sqrt{\frac{ib/2}{\gamma +i({\omega }_{0}-\omega )}}$$

The real part of ${\xi }_{0}$ takes an especially simple form at the band edge

21 $${\xi }_{0}=\mathrm{Re}[\xi ({\omega }_{0})]=\sqrt{b/\gamma }=\sqrt{b{\tau }_{r}}$$

which we call the *nonlocality length*, the characteristic distance that light travels in-plane before scattering out when engaging with the band-edge mode (a quantitative measure of the nonlocality of the metasurface). Since nonlocality is equivalently defined as a dependence on the incident wavevector, no optical interface is truly local. However, as a rule of thumb, if the nonlocality length is larger than the wavelength in the surrounding media, the resulting device may be considered nonlocal, since its nonlocality cannot be neglected (see Supplementary Section S4 for a comparison of a nonlocal metasurface to a bare interface between two media).

### Physical constraints

Before proceeding with the application of STCMT to practical metasurface devices, we must determine the relevant physical constraints to our system, including time-reversal invariance, reciprocity and conservation of energy. In TCMT, these constraints impose the well-known requirements^5^

22 $$\langle d|d\rangle =2\gamma$$

23 $$|\kappa \rangle =|d\rangle$$

24 $$C|{d}^{\ast }\rangle =-|d\rangle$$

These results hold, for instance, in infinitely periodic devices supporting q-BICs (see Supplementary Section S1). As detailed in Supplementary Section S5, we find that in our STCMT all these conditions hold *locally*, i.e.,

25 $$\langle d(x)|d(x)\rangle =2\gamma$$

26 $$|\kappa (x)\rangle =|d(x)\rangle$$

27 $$C(x)|{d}^{\ast }(x)\rangle =-|d(x)\rangle$$

We stress that this result is not obvious a priori and, indeed, these do not hold for the general case described by Eqs. (6) and (7). Future work may explore systems with nonlocal coupling (i.e., devices for which Eq. (8) do not hold), in which case Eqs. (26) and (27) are no longer valid.

The significance of this result for modeling diffractive nonlocal metasurfaces should not go underappreciated, as it confers a major simplification to model this class of systems and enables the introduction of a semi-analytical model to describe them. Because the *coupling* is local, each unit cell of a nonlocal metasurface may be modeled as if it were in an infinite array, allowing easy correlation between geometric and phenomenological degrees of freedom (compatible with TCMT in the momentum-frequency domain). Then, a given space-varying nonlocal metasurface is treated by specifying a spatial profile of the coupling coefficients, wherein the correlations across the surface are captured by a shift-invariant Green’s function (*modal* nonlocality). This design flow is compatible with the foundational design principles of *local* metasurfaces^43^, wherein a library of pre-computed optical scatterers may be arrayed across the surface as if they operate independently of their nearest neighbors. This ubiquitous design approach is sometimes called the “local phase approximation”. Yet, we find that, when Eq. (8) holds, the same design principles are applicable even in the much more complex scenario discussed in this work, considering *nonlocal* responses due to a guided mode.

### Eigenwaves

Of central interest to nonlocal metasurfaces is the principal eigenwave, which is the wavefront that a nonlocal metasurface selects for: maximal reflectance is achieved when the eigenwave is incident, and the reflected wave is the eigenwave’s phase conjugated copy [55]. Using $\rho ={\sigma }_{11}$ as the reflection kernel for light incident from side 1, the eigenwave from side 1 is solution to the relation

28 $${E}_{eig}^{\ast }(x,\omega )=\int {\rm{d}}x^{\prime} \rho (x,x^{\prime} ,\omega ){E}_{eig}(x^{\prime} ,\omega )$$

or equivalently

29 $${E}_{eig}(x,\omega )=\int {\rm{d}}x^{\prime} {\rho }^{\ast }(x,x^{\prime} ,\omega ){E}_{eig}^{\ast }(x^{\prime} ,\omega )$$

Combing Eqs. (28) and (29) we find

30 $${E}_{eig}(x,\omega )=\int {\rm{d}}x^{\prime} \int {\rm{d}}x^{\prime\prime} {\rho }^{\ast }(x,x^{\prime} ,\omega )\rho (x^{\prime} ,x^{\prime\prime} ,\omega ){E}_{eig}(x^{\prime\prime} ,\omega )$$

suggesting that the eigenwave of interest is the eigenvector of

31 $${R}_{eig}{E}_{eig}(x,\omega )=\int {\rm{d}}x^{\prime} \int {\rm{d}}x^{\prime\prime} {\rho }^{\ast }(x,x^{\prime} ,\omega )\rho (x^{\prime} ,x^{\prime\prime} ,\omega ){E}_{eig}(x^{\prime\prime} ,\omega )$$

that has the largest eigenvalue ${R}_{eig}$. We will show that STCMT can be used to explicitly calculate the eigenwaves of a given nonlocal metasurface. Equation (31) also implies the existence of higher-order eigenwaves that reflect to their time-reversed copy but with non-maximal reflectance and eigenwaves for frequencies other than the band-edge frequency ${\omega }_{0}$.

### Diffractive nonlocal metasurfaces

**Fig. 2:**
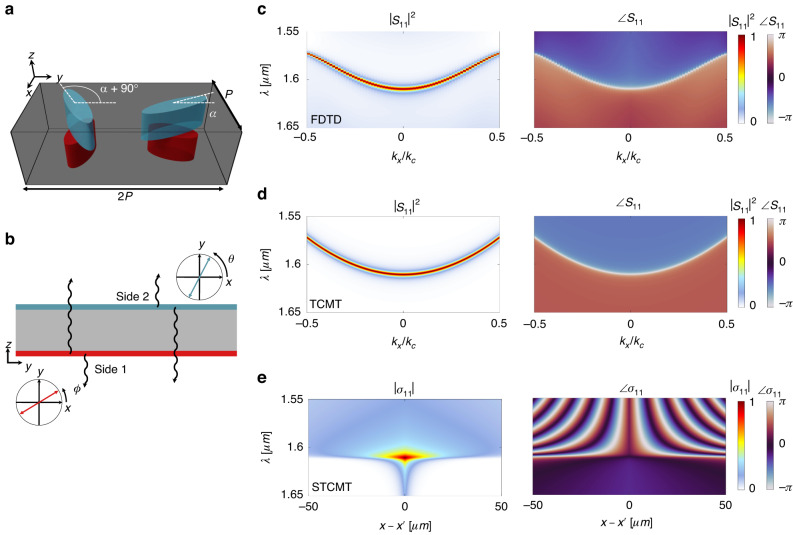
Modeling periodic nonlocal metasurfaces with FDTD, TCMT, and STCMT. **a** Prototypical unit cell for a diffractive nonlocal metasurface, containing four elliptical inclusions. The orientation angle $\alpha$ is the key parameter controlling the geometric phase (the orientation angles of the bottom (red) ellipses are not independent of $\alpha$). The lattice parameter $\rm{a}$ satisfies $2P < {\lambda }_{0}$, eliminating all but 1 diffraction order on each side. **b** Schematic of the coupled mode theory approximation of the scattering in (a), wherein independent scattering events occur at the interfaces, each of which may generally couple to both side 1 (down) and side 2 (up). **c** Reflectance and reflection phase in the momentum-frequency response of an example meta-unit, computed by Finite Difference Time Domain (FDTD). **d** Reflectance and reflection phase in the momentum-frequency response of an example meta-unit, modeled using TCMT. **e** Amplitude and phase of ${\sigma }_{11}$ in the space-frequency domain of the same meta-unit, a key object for modeling this system using STCMT

In this section, we apply STCMT to illustrative examples, confirming that our analytical framework reproduces the results of full-wave simulations, quantitatively captures the underlying physics and enables rational designs for relevant functionalities. Schematically depicted in Fig. 2a, we explore systems based on refs. ^33^ and^34^, composed of four elliptical inclusions controlling the scattering (polarization angles $\phi$ and $\theta$) at the top and bottom interfaces simultaneously and independently [Fig. 2b]. Figure 2c, d shows the calculated reflectance and reflected phase for an example meta-unit based on this system, computed using the finite difference time domain (FDTD) method. Figure 2e, f depict the TCMT modeling of the infinitely periodic meta-unit (developed in Supplementary Section S1b), with (in units such that the speed of light is ${c}_{0}=1$) the band edge frequency ${\omega }_{0}=2\pi /{\lambda }_{be}$ with ${\lambda }_{be}=1.6104\,\mu m$, the band curvature $b=0.021\,\mu m$, and the lifetime ${\tau }_{r}=250\mu m$. In particular, we use the chiral q-BIC system in refs. ^33^ and^34^, in which the response was shown to: (i) have near-zero background reflection, (ii) exclusively engage circularly polarized light of one handedness, and (iii) decay with a geometric phase of $2\alpha$ for a meta-unit characterized by in-plane orientation angle $\alpha$. In a scalar basis of the selected spin we have

32 $$|d(x)\rangle =i\sqrt{\gamma }\left[\begin{array}{c}\exp [i2\alpha (x)]\\ -i\exp [-i2\alpha (x)]\end{array}\right]$$

where the first element is the coefficient scattering to side 1 and the second element is the coefficient scattering to side 2. For a system based on the meta-units in Fig. 2 (and refs. ^33,34^), this yields [from Eqs. (18) and (19)]

33 $$\begin{array}{ll}\sigma (x,x^{\prime} ,\omega )=\delta (x-x^{\prime} )\left[\begin{array}{cc}0 & -i\\ -i & 0\end{array}\right]+i\frac{\gamma }{b}\xi (\omega )\exp\\ \left(-\frac{|x-x^{\prime} |}{\xi (\omega )}\right)\left[\begin{array}{cc}{e}^{i2\alpha (x)}{e}^{i2\alpha (x^{\prime} )} & -i{e}^{i2\alpha (x)}{e}^{-i2\alpha (x^{\prime} )}\\ -i{e}^{-i2\alpha (x)}{e}^{i2\alpha (x^{\prime} )} & -{e}^{-i2\alpha (x)}{e}^{-i2\alpha (x^{\prime} )}\end{array}\right]\end{array}$$

For clarity, we detail the behavior for light incident from side 1, separating the $\sigma$ into a complex reflection kernel $\rho ={\sigma }_{11}$ and a complex transmission kernel $\tau ={\sigma }_{21}$:

34 $$\begin{array}{ll}\rho (x,x^{\prime} ,\omega )=i\frac{\gamma }{b}\xi (\omega )\exp \left(-\frac{|x-x^{\prime} |}{\xi (\omega )}\right){e}^{i2\alpha (x)}{e}^{i2\alpha (x^{\prime} )}\\ \tau (x,x^{\prime} ,\omega )=-i\delta (x-x^{\prime} )+\frac{\gamma }{b}\xi (\omega )\exp \left(-\frac{|x-x^{\prime} |}{\xi (\omega )}\right){e}^{-i2\alpha (x)}{e}^{i2\alpha (x^{\prime} )}\end{array}$$

The other kernels ${\sigma }_{12}$ and ${\sigma }_{22}$ are treated equivalently. Figure 2g, h shows the magnitude and phase of ${\sigma }_{11}$ for the meta-unit corresponding to Fig. 2e, f. Note that in this case the phase angle $\alpha (x)=\alpha$ is constant, meaning that this metasurface can be handled with conventional TCMT. However, in the following examples, the angle is spatially varied and TCMT is not suitable.

### Linear phase gradient: analytical model

An archetypal metasurface functionality is anomalously deflection using a linear phase gradient. Here, we consider a gradient in the geometric angle following

35 $$2\alpha (x)=-{k}_{G}x$$

In this case, the nonlocal kernels take the form

36 $$\begin{array}{ll}\rho (x,x^{\prime} ,\omega )=i\frac{\gamma }{b}\xi (\omega )\exp \left(-\frac{|x-x^{\prime} |}{\xi (\omega )}\right){e}^{-i{k}_{G}x}{e}^{-i{k}_{G}x^{\prime} }\\ \tau (x,x^{\prime} ,\omega )=-i\delta (x-x^{\prime} )+\frac{\gamma }{b}\xi (\omega )\exp \left(-\frac{|x-x^{\prime} |}{\xi (\omega )}\right){e}^{i{k}_{G}x}{e}^{-i{k}_{G}x}\end{array}$$

When ${k}_{G}=0$, we recover the nonlocal reflection kernel without phase gradient, which we denote with the subscript $0$:

37 $$\begin{array}{ll}{\rho }_{0}(x,x^{\prime} ,\omega )=i\frac{\gamma }{b}\xi (\omega )\exp \left(-\frac{|x-x^{\prime} |}{\xi (\omega )}\right)\\ {\tau }_{0}(x,x^{\prime} ,\omega )=-i\delta (x-x^{\prime} )+\frac{\gamma }{b}\xi (\omega )\exp \left(-\frac{|x-x^{\prime} |}{\xi (\omega )}\right)\end{array}$$

Since a phase gradient provides a spatially constant momentum kick, it is natural to study these devices in momentum space. In particular, we seek the scattering matrix

38 $$S(k,k^{\prime} ,\omega )=\left[\begin{array}{cc}{S}_{11}(k,k^{\prime} ,\omega ) & {S}_{12}(k,k^{\prime} ,\omega )\\ {S}_{21}(k,k^{\prime} ,\omega ) & {S}_{22}(k,k^{\prime} ,\omega )\end{array}\right]$$

where $k^{\prime}$ and $k$ are the wavevectors of the incoming and outgoing plane waves, respectively; the indices refer to the top and bottom sides of the metasurface. We then relate this scattering matrix to $\sigma$ through the mixed Fourier transform (see Supplementary Section S6)

39 $${S}_{ij}(k,k^{\prime} ,\omega )=\int {\rm{d}}x\int {\rm{d}}x^{\prime} {\sigma }_{ij}(x,x^{\prime} ,\omega )\exp (ik^{\prime} x^{\prime} )\exp (-ikx)$$

Using this relation, the scattering components for a metasurface without phase gradient are

40 $$\begin{array}{ll}{S}_{11}^{0}(k,k^{\prime} ,\omega )=\frac{-\delta (k-k^{\prime} )}{1-i\left(\omega -{\omega }_{0}-\frac{b}{2}{k}^{2}\right){\tau }_{r}}\\ {S}_{21}^{0}(k,k^{\prime} ,\omega )=\delta (k-k^{\prime} )\left[-i+\frac{i}{1-i(\omega -{\omega }_{0}-\frac{b}{2}{k}^{2}){\tau }_{r}}\right]\\ {S}_{22}^{0}(k,k^{\prime} ,\omega )={S}_{11}^{0}(k,k^{\prime} ,\omega )\\ {S}_{12}^{0}(k,k^{\prime} ,\omega )={S}_{21}^{0}(k,k^{\prime} ,\omega )\end{array}$$

Notably, this result is identical to conventional TCMT but with the explicit addition of a Dirac $\delta$ term enforcing scattering only when $k=k^{\prime}$ (conservation of momentum in a specular process).

Meanwhile, the scenario with nonzero ${k}_{G}$ in terms of Eq. (37) yields

41 $$\begin{array}{c}\rho (x,x^{\prime} ,\omega )={\rho }_{0}(x-x^{\prime} ,\omega ){e}^{-i{k}_{G}x}{e}^{-i{k}_{G}x^{\prime} }\\ \tau (x,x^{\prime} ,\omega )={\tau }_{0}(x-x^{\prime} ,\omega ){e}^{i{k}_{G}x}{e}^{-i{k}_{G}x^{\prime} }\end{array}$$

which implies, due to the basic properties of the Fourier transform,

42 $$\begin{array}{c}{S}_{11}(k,k^{\prime} ,\omega )={S}_{11}^{0}(k+{k}_{G},k^{\prime} -{k}_{G},\omega )\\ {S}_{21}(k,k^{\prime} ,\omega )={S}_{21}^{0}(k-{k}_{G},k^{\prime} -{k}_{G},\omega )\\ {S}_{22}(k,k^{\prime} ,\omega )={S}_{11}^{0}(k-{k}_{G},k^{\prime} +{k}_{G},\omega )\\ {S}_{12}(k,k^{\prime} ,\omega )={S}_{12}^{0}(k+{k}_{G},k^{\prime} +{k}_{G},\omega )\end{array}$$

Hence, our first conclusion from STCMT is that the impact of a nonlocal phase gradient is to shift the scattering matrices in *k*-space, in both the *input* and *output* spaces. In particular, in the reflection elements [${S}_{11}(k,k^{\prime} ,\omega )$ and ${S}_{22}(k,k^{\prime} ,\omega )$] the shift in input and output momentum is equal and opposite, producing anomalous reflection. Since we have nonzero scattering only when the Dirac delta function’s argument is 0, from side 1 we see that reflection occurs only when $k=k^{\prime} +2{k}_{G}$, and from side 2 only when $k=k^{\prime} -2{k}_{G}$. Hence, our results capture the fact that the anomalous reflection is associated with the $m=\pm 2$ diffraction orders^31^, and the direction of momentum kick imparted by the nonlocal metasurface depends the direction of incidence^28^.

### Linear phase gradient: numerical simulations

We now validate our analytical model numerically for ${k}_{G}=2\pi /W$ and $W=6.4\,\mu m$, focusing here on the band-edge frequency (Supplementary Section S7 studies frequencies off the band edge). It is useful here to cast STCMT in matrix form, detailed in Supplementary Section S8, which makes the scattering kernels discrete. We write them as ${\rm P}$ and ${\rm T}$ (capital $\rho$ and $\tau$) and the input electric field as a column vector ${{\bf{E}}}_{in}$, where each entry is the field at a discretized position $x$, and we compute the reflected ${{\bf{E}}}_{r}$ and transmitted ${{\bf{E}}}_{t}$ fields with

43 $$\begin{array}{c}{{\bf{E}}}_{r}={\rm P}{{\bf{E}}}_{in}\\ {{\bf{E}}}_{t}={\rm T}{{\bf{E}}}_{in}\end{array}$$

The main diagonal of ${\rm P}$ and ${\rm T}$ correspond to $x=x^{\prime}$, i.e., the local response, while the off-diagonal components contain the correlations across distant positions, i.e., the nonlocal response. Finally, given the finite nature, boundary conditions must be specified; Supplementary Section S9 details both periodic and radiative boundary conditions. Supplementary Section S10 describes the extension of this procedure to 2D metasurfaces.

**Fig. 3:**
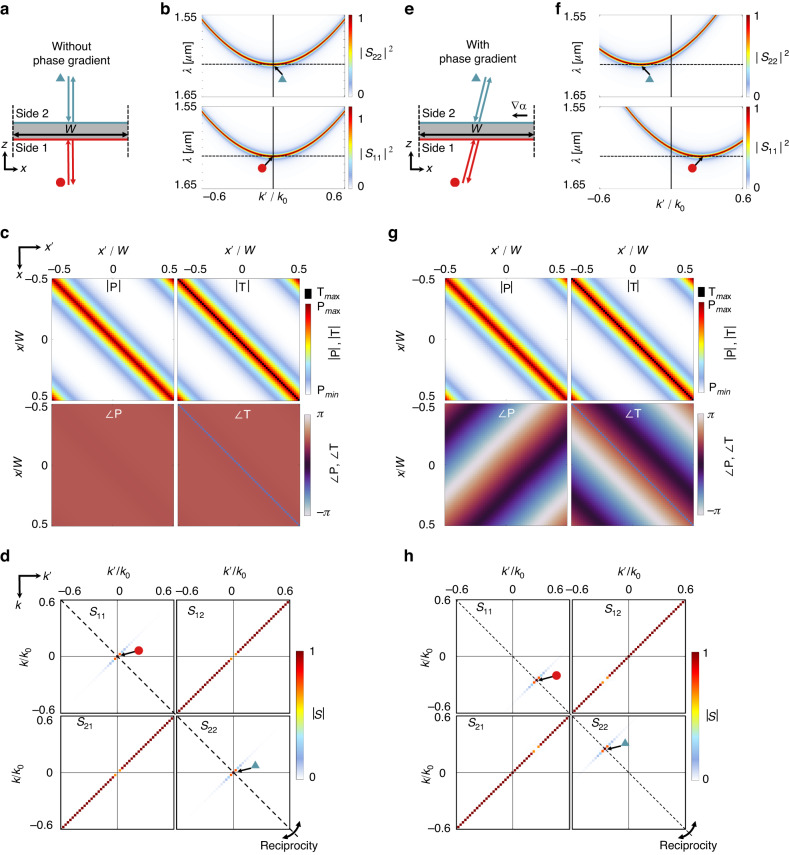
Analysis of phase gradient devices. **a** Schematic of the resonant response of a nonlocal phase gradient device with ${k}_{G}=0$ at the band-edge frequency. **b** Reflectance maps of ${S}_{11}$ and ${S}_{22}$ as a function of incident momentum $k^{\prime}$ for the device in (**a**), where the dashed line indicates the band-edge frequency. **c** Nonlocal kernel matrices for the device in (**a**) at the band-edge frequency (note that ${\rm T}$ is identical to ${\rm P}$ except along the main diagonal, where its magnitude is much higher). **d** Scattering matrix for the device in (**a**), showing unity specular reflectance only at normal incidence. **e**–**h** Same as in (**a**–**d**) but for a device with ${k}_{G}=2\pi /W$ operated at the band-edge frequency. Note that for sufficient resolution, (**d**, **h**) are computed using the mixed Fast Fourier Transform of a version of (**c**, **g**) including 10 metasurface periods. The red circle and blue triangle track the band-edge response from below and above respectively, in each case

Using this discretized form of STCMT, Fig. 3 compares the scenario without a phase gradient [Fig. 3a–d] to the case with a phase gradient [Fig. 3e–h]. The former is identical to the structures described in Fig. 2., while the latter differs only in that the orientation angles of the ellipses varies spatially. Figure 3a schematically shows resonant reflection at the band-edge frequency, $\omega ={\omega }_{0}$, of the typical parabolic band diagrams in Fig. 3b, depicting specular reflection at normal incidence when incident from either side (${S}_{11}$ and ${S}_{22}$). Figure 3c shows the corresponding amplitude and phase of ${\rm P}$ and ${\rm T}$, which, using Eq. (39), yield the scattering matrix elements in Fig. 3d. In this case, specular transmission arises except at normal incidence from either side, wherein specular reflection occurs (tracked by the red circle and blue triangle from sides 1 and 2, respectively).

In contrast, in the presence of a phase gradient the device retroreflects light at the resonance frequency, as shown in Fig. 3c. From side 1, the band edge mode is shifted in k-space by ${k}_{G}$, meaning that the resonance only occurs for $k^{\prime} ={k}_{G}$:

44 $$\omega (k^{\prime} )={\omega }_{0}+\frac{b}{2}{(k^{\prime} -{k}_{G})}^{2}$$

The shift is in the opposite direction for light from side 2 [Fig. 3f]. This shift, as well as the anomalous reflection, is encoded in ${\rm P}$ and ${\rm T}$ within the phase information [Fig. 3g], and the corresponding scattering matrix at the band-edge frequency [Fig. 3h] confirms that the resonant mode occurs at $k^{\prime} ={k}_{G}$ and $k=-{k}_{G}$ when light is incident from side 1 (red circle), while it occurs at $k^{\prime} =-{k}_{G}$ and $k={k}_{G}$ when light is incident from side 2 (blue triangle). Note that this behavior is consistent with the requirements stemming from reciprocity: the scattering matrices must be symmetric about the main diagonal, which corresponds to $k=-k^{\prime}$, or retroreflection (see also Supplementary Section S5). Hence, we see that the action of the phase gradient is to shift the band-edge response along the retroreflection condition by an amount $({k}_{G},-{k}_{G})$ in ${S}_{11}$. The corresponding shift in ${S}_{22}$ is $(-{k}_{G},{k}_{G})$, consistent with conservation of energy [the dip and peak occurring at the same $k^\prime$ in *S*_12_ and *S*_22_, and likewise for *S*_11_ and *S*_21_]. That is, the behavior of a nonlocal phase gradient is to *tilt* the response in *k*-space [Fig. 3e] rather than add a unidirectional momentum kick irrespective of incident direction: it is a vertically asymmetric phenomenon, therefore requiring vertically asymmetry in the structure to achieve, irrespective of polarization^28,44^. The scattered fields corresponding to the peaks in (h) are shown in Fig. S4 of the supplementary materials.

### Nonlocal metalens

**Fig. 4:**
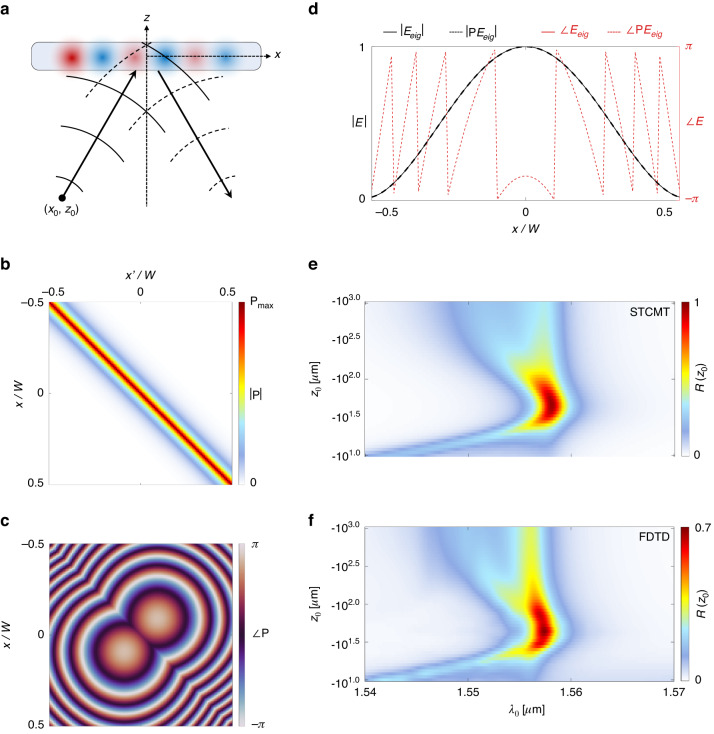
Nonlocal metalens. **a** Nonlocal metalens selective to a point source placed at $({x}_{0},{z}_{0})$. Magnitude (**b**) and phase (**c**) of the nonlocal reflection kernel ${\rm P}$ for a metalens of width $W=50\,\mu m$ with q-BIC parameters $b=0.032\,\mu m$,${\lambda }_{0}=1.558\,\mu m$, and ${\tau }_{r}=297\,\mu m$, and ${\rm{NA}}=0.48$, matched to the system in ref. ^34^. **d** Eigenwave and reflected wave, showing unchanged amplitude and conjugated phase profiles upon reflection. The reflectance due to excitation by point sources placed at locations ${z}_{0}$ on the optical axis $(x,y)=(0,0)$ for (**e**) STCMT model and (**f**) full-wave simulations based on the system reported in ref. ^34^

Next, we treat nonlocal metalenses—an archetypal example of spatially varying nonlocal metasurfaces of broad relevance for applications [Fig. 4a]. We first analytically study the eigenwaves of nonlocal metalenses, and then numerically demonstrate agreement with the spatial selectivity demonstrated in ref. ^34^. Our validation with full-wave simulations then motivates us to employ STCMT to rapidly and thoroughly study a variety of nonlocal metalenses, shedding light on their operation. In particular, we demonstrate that, as a function of the band structure $b$, the Q-factor $Q={\omega }_{0}{\tau }_{r}$, and the numerical aperture of the metalens NA, a nonlocal metalens can transition between two regimes of operation: wavefront-selective and wavefront-shaping. Supplementary Section S11 extends the following to frequencies off the band edge.

In particular, we study nonlocal metalenses described by

45 $$2\alpha (x) =-{k}_{0}\sqrt{{x}^{2}+{f}^{2}}$$

Using relation (31), the eigenwave of the metalens is given by

46 $$\begin{array}{ll}{R}_{eig}{E}_{eig}(x,\omega )={\int }_{\!-W/2}^{W/2}{\rm{d}}x^{\prime} {\int }_{\!-W/2}^{W/2}{\rm{d}}x^{\prime\prime} {\rho }_{0}^{\ast }(x-x^{\prime} ,\omega )\\{\rho }_{0}(x^{\prime} -x^{\prime\prime} ,\omega ){e}^{i{k}_{0}\sqrt{{x}^{2}+{f}^{2}}}{e}^{-i{k}_{0}\sqrt{{(x^{\prime\prime} )}^{2}+{f}^{2}}}{E}_{eig}(x^{\prime\prime} ,\omega )\end{array}$$

By inspection, as $W\to \infty$ (i.e., ignoring boundary effects), we have

47 $${E}_{eig}(x,\omega )={e}^{i{k}_{0}\sqrt{{x}^{2}+{f}^{2}}}$$

because in this case

48 $${R}_{eig}={\int }_{\!-W/2}^{W/2}{\rm{d}}x^{\prime} {\int }_{\!-W/2}^{W/2}{\rm{d}}x^{\prime\prime} {\rho }_{0}^{\ast }(x-x^{\prime} ,\omega ){\rho }_{0}(x^{\prime} -x^{\prime \prime} ,\omega )=1$$

which is identical to the spatially invariant q-BIC modeled by the subscripted kernels. For finite $W$ and radiative boundaries, Eq. (48) does not yield a perfectly unity result—to be expected for a finite device (see Supplementary Section S9). Nevertheless, the idealized solution^45^ captures the underlying phenomenon: this nonlocal metalens is selective to a point source set at ${z}_{0}=-f$, just as described in ref. ^34^.

However, unlike ref. ^34^, here we may specify the non-ideal eigenwaves for finite structures, including radiative boundary conditions, e.g., using the discrete matrix form of our STCMT. The nonlocal reflection matrix for this scenario is seen in Fig. 4b, c, from which the eigenwave ${E}_{eig}$ is computed, depicted in Fig. 4d overlaid with the reflected wave (${\rm P}{E}_{eig}$), which is the phase conjugated copy of the input as expected. We also may numerically compute the reflectance due to point sources incident from side 1 at position $({x}_{0},{z}_{0})$ [Fig. 4a]:

49 $${E}_{in}(x,\omega )=\exp \left(i{k}_{0}\sqrt{{(x-{x}_{0})}^{2}+{z}_{0}^{2}}\right)$$

Using the metasurface introduced in ref. ^34^, we numerically compute the reflectance Fig. 4e as a function of wavelength and ${z}_{0}$ (while keeping ${x}_{0}=0$) and find remarkable agreement with the results from full-wave simulation in Fig. 4f. The peak reflectance in the STCMT is near-unity, while full-wave simulations predict peak reflectance near $70 \%$, suggesting that the metalens has room for optimization in its design. Regardless, our STCMT can clearly capture the response of the nonlocal metalens.

**Fig. 5:**
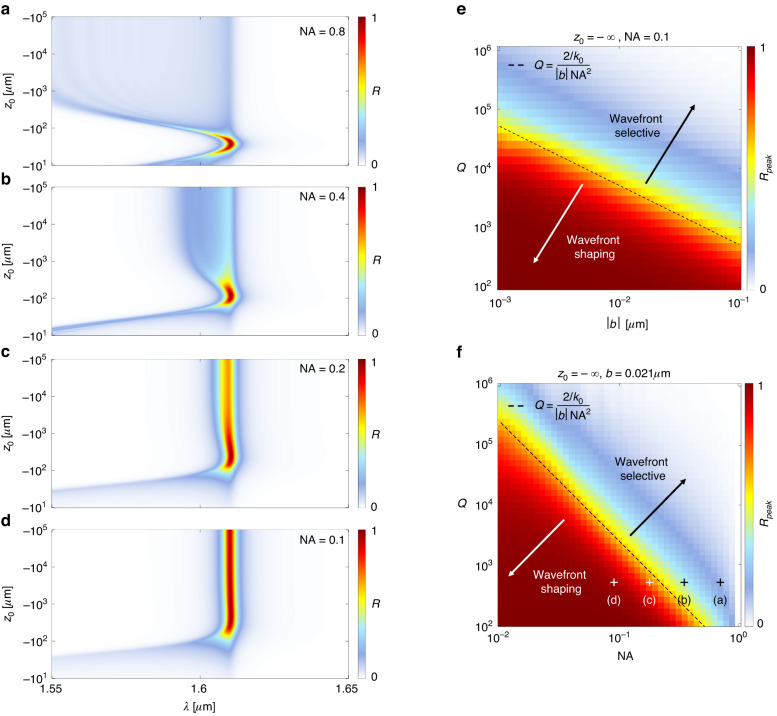
Selectivity of a nonlocal metalens. **a**–**d** Reflectance for a device with $W=75\,\mu m$ and varying ${\rm{NA}}$, with q-BIC parameters $b=0.021\,\mu m$, ${\lambda }_{0}=1.6104\,\mu m$, and ${\tau }_{r}=250\,\mu m$, due to excitation by point sources placed at locations ${z}_{0}$ on the optical axis $(x,y)=(0,0)$. **e** Peak reflectance due to a normally incident plane wave for a device with ${\rm{NA}}=0.1$ as a function of $b$ and $Q$. **f** Peak reflectance due to a normally incident plane wave for a device with ${\rm{b}}=0.021\,\mu m$ as a function of ${\rm{NA}}$ and $Q$

Next, we explore the response of the system in Fig. 2 as a function of $b$, $Q$, and numerical aperture, ${\rm{NA}}=n\frac{W/2}{\sqrt{{(W/2)}^{2}+{(f/2)}^{2}}}$, where $n$ is the index of refraction of the surrounding medium. The ${\rm{NA}}$ characterizes the range of deflection angles encoded into the q-BIC, and hence it also characterizes the range of resonant wavelengths across the device for normally incident light. As the ${\rm{NA}}$ increases, the part of the nonlocal metalens resonating at a given frequency is reduced, and the reflectance also diminishes—i.e., spatial selectivity increases with ${\rm{NA}}$. This feature is confirmed in Fig. 5a–d, which show the spectral reflectance as a function of ${z}_{0}$ for four values of ${\rm{NA}}$. When ${\rm{NA}}=0.8$, representing a very large range of deflection angles, only point sources placed near ${z}_{0}=-f$ reflect substantial power at the band-edge frequency. That is, point sources near the eigenwave experience large reflectivity, while point sources away from the eigenwave are non-resonantly transmitted. When the ${\rm{NA}}$ lowers, we observe an increase in reflectance away from the eigenwave condition, e.g., for large $|{z}_{0}|$, which approaches a plane wave in the limit that ${z}_{0}\to -\infty$. So while when ${\rm{NA}}=0.8$ we have a wavefront-selective device, in contrast, when ${\rm{NA}}=0.1$, we have a wavefront-shaping device, abiding large reflectance under a larger range of incident conditions.

Importantly, the transition from wavefront-selective to wavefront-shaping is a function of the degree of locality. When ${\xi }_{0}$ is substantially larger than ${\lambda }_{0}$, the device is highly selective, i.e., it only acts as a wavefront-shaping device for very low ${\rm{NA}}$. But when ${\xi }_{0}$ is comparable to ${\lambda }_{0}$, the device is not selective, i.e., it acts as a wavefront-shaping device for a large range of ${\rm{NA}}$. We find (Supplementary Section S12) that the wavefront-shaping regime is satisfied when

50 $${\rm{NA}} < \sqrt{\frac{2/{k}_{0}}{|b|Q}}=\frac{1}{\sqrt{2}\pi }\frac{{\lambda }_{0}}{{\xi }_{0}}$$

We therefore may quantitatively conclude that the degree of spatial selectivity is due to the degree of nonlocality of the q-BIC. Supplementary Section S13 further quantifies this feature by discussing the expansion in the eigenbasis of the device, an analysis related to modal expansions in optics^46^.

We next explore devices with varying $b$, $Q$, and ${\rm{NA}}$ to demonstrate the transition between wavefront-selective and wavefront-shaping regimes. Figure 5e shows the peak reflectance for a normally incident plane wave ${z}_{0}=-\infty$ for a device with ${\rm{NA}}=0.1$ as a function of $b$ and $Q$: when they are both large (a highly nonlocal device), the plane wave is transmitted unperturbed through the device, indicating wavefront-selectivity. When they are both small (a highly local device), the plane wave is anomalously reflected with near-unity efficiency, and the device is wavefront-shaping. The transition Q-factor for a given value of $b$ is overlaid on Fig. 5e by rearranging Eq. (50), showing good agreement between the numerical calculations and the physical arguments outlined here. Additionally, Fig. 5f shows a device with $b=0.021\,\mu m$ as a function of ${\rm{NA}}$ and $Q$, showing similar agreement in the transition between the two regimes.

### Thermal metasurfaces

To further showcase the applicability of STCMT, we now model thermal metasurfaces^35,44^, which provide a powerful platform to shape emission from metasurfaces. STCMT enables rapid study and prototyping of focused incoherent light emission with respect to ${\xi }_{0}$ and the lens design parameters. To tackle this problem, we extend the STCMT formulation by (i) accounting for polarization ports in a reflection- and absorption-only scattering problem, (ii) incorporating a spatially varying local metasurface response in the background $C(x)$, and (iii) adding absorption loss, distinguishing the radiative scattering rate ${\gamma }_{r}$ and the nonradiative scattering (loss) rate ${\gamma }_{nr}$. Here, the scattering matrix ${S}_{ij}$ is indexed by linear polarizations $i,j\in x,y$, and the background scattering matrix has full birefringence with a fast axis oriented at $\theta (x)$:

51 $$C(x)=\left[\begin{array}{cc}\cos [2\theta (x)] & \sin [2\theta (x)]\\ \sin [2\theta (x)] & -\,\cos [2\theta (x)]\end{array}\right]$$

Meanwhile, for perturbations governing the q-BIC defined by an orientation angle $\alpha$, the coupling coefficients are^44^

52 $$|d(x)\rangle =\sqrt{{\gamma }_{r}}\left\{\left[\begin{array}{c}\cos [2\alpha (x)]\\ \sin [2\alpha (x)]\end{array}\right]-i\left[\begin{array}{c}\cos [2\theta (x)-2\alpha (x)]\\ \sin [2\theta (x)-2\alpha (x)]\end{array}\right]\right\}$$

Finally, to account for the presence of loss, we make the simple adjustment

53 $$\xi (\omega )=\sqrt{\frac{ib/2}{{\gamma }_{r}+{\gamma }_{nr}+i({\omega }_{0}-\omega )}}$$

For clarity, we may also separate the scattering kernel into its local and nonlocal components

54 $$\sigma (x,x^{\prime} ,\omega )={\rho }_{loc}(x)+{\rho }_{nonloc}(x,x^{\prime} ,\omega )$$

where

55 $${\rho }_{loc}(x)=C(x)\delta (x-x^{\prime} )$$

56 $${\rho }_{nonloc}(x,x^{\prime} ,\omega )=G(x,x^{\prime} ,\omega )|d(x)\rangle \langle {d}^{\ast }(x^{\prime} )|$$

Then, assuming that the power not reflected is absorbed by the device, the absorption of a given input wave can be simply computed as

57 $${\mathrm{A}}(\omega )=1-\langle {s}_{+}|{\sigma }^{\ast }(x,x^{\prime} ,\omega )\sigma (x,x^{\prime} ,\omega )|{s}_{+}\rangle$$

while the corresponding thermal emission is described by the time-reversed wave, following the universal modal radiation laws^47^.

**Fig. 6:**
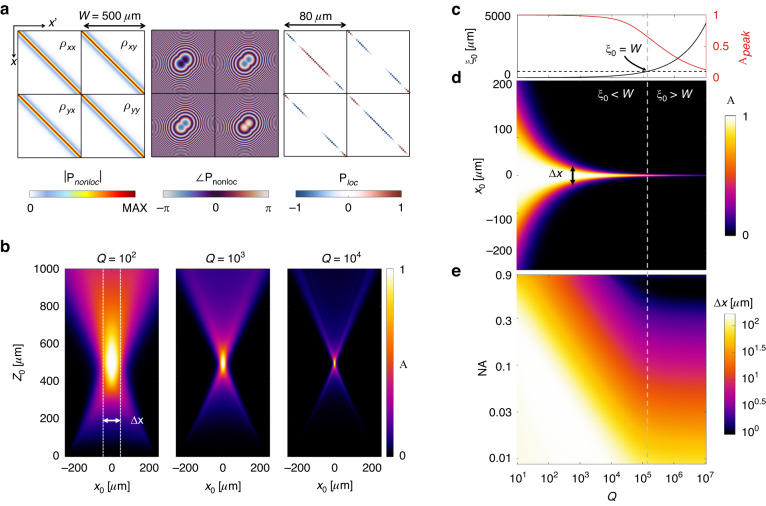
Thermal metalenses. **a** Components of the nonlocal reflection kernel for a thermal metalens. (left) Magnitude and (middle) phase of the nonlocal component of the nonlocal reflection kernel ${{\rm P}}_{nonloc}$ at the band edge frequency of a thermal metalens that is $W=500\,\mu m$ wide, focusing to a point $f=500\,\mu m$ away with ${\lambda }_{0}=4\,\mu m$. (Right) The corresponding local reflection kernel ${{\rm P}}_{loc}$ (with only the central $80\,\mu m$ depicted for visual clarity). **b** Computed absorption for sample point sources placed at positions $({x}_{0},{z}_{0})$ due to a thermal lens with $b=0.15\,\mu m$ for three example Q-factors. **c** Nonlocality ${\xi }_{0}=\sqrt{b{\tau }_{r}}$ and peak absorption ${{A}\,}_{peak}$ [i.e., for a source placed at $({x}_{0},{z}_{0})=(0,f)$] as a function of $Q$. The horizontal black dashed line marks a length of $W$; the vertical gray dashed line marks the $Q$ at which ${\xi }_{0}=W$. **d** Absorption for point sources placed in the focal plane at ${z}_{0}=f$ as a function of $Q$. **e** Full width at half maximum $\varDelta x$ of the response at the focal plane as a function of $Q$ and numerical aperture $NA$, showing two regimes of behavior

As an archetypal example, Fig. 6 studies a thermal metalens that preferentially emits to a focal point $f$ with a circular polarization, encoded using the profiles^35^

58 $$\theta (x)=\pm \frac{2\pi }{{\lambda }_{0}}\sqrt{{x}^{2}+{f}^{2}}$$

59 $$2\alpha (x)=\theta (x)\pm \frac{\pi }{4}$$

for the chosen polarization $$[\begin{array}{cc}1 & \mp \end{array}i]/\sqrt{2}$$. Figure 6a shows the nonlocal and local scattering kernels, where the four quadrants correspond to the input and output polarization states in an $x,y$ basis. In contrast to the lossless, scalar nonlocal metalens of the previous section, the local kernel in Fig. 6a encodes a varying birefringent axis along the main diagonals of each quadrant [Eq. (51) with Eq. (58)]. Then, we compute the absorption of the metalens due to excitation by point sources placed at positions $({x}_{0},{z}_{0})$ above the metasurface. Figure 6b shows the spatial absorption maps for three example Q-factors operating at the band-edge frequency, showing that the focal spot tightens as the lifetime of the q-BIC grows. This directly corresponds to an increase in the nonlocality length ${\xi }_{0}=\sqrt{b{\tau }_{r}}$, i.e., the metasurface becomes increasingly wavefront selective; hence, increasing $b$ has the same effect at the band-edge frequency. Supplementary Section S14 discusses the response as a function of frequency.

We next rapidly compute the full width at half maximum (FWHM), $\varDelta x$ of the thermal metalens response as a function of Q-factor and $NA$. Figure 6c shows how ${\xi }_{0}$ and the peak absorption ${A\,}_{peak}$ depend on $Q$. Notably, ${A\,}_{peak}$ drops off around ${\xi }_{0}=W$, i.e., when the nonlocality length is comparable to the aperture size (width) of the lens. This stems from incomplete interference: if the finite size of the lens is too small, the q-BIC leaks out the sides of the metalens instead of building up a complete Fano resonance. Figure 6d shows the absorption at the focal plane ${z}_{0}=f$ as a function of $Q$, showing a continuous trend consistent with Fig. 6d when ${\xi }_{0} < W$. However, when ${\xi }_{0} > W$ we notice instead that the FWHM plateaus above a certain $Q$ [Fig. 6e]. Below that Q-factor, $\varDelta x$ is determined by the target $NA$ together with $Q$; above that Q-factor, $\varDelta x$ is determined only by the $NA$. From this, we learn that there is a threshold $Q$ above which the focal spot does not continue to tighten; instead, increasing the Q-factor only reduces the intensity of the response. These results suggest, intuitively, that to improve the response further (i.e., reduce $\varDelta x$ while maintaining *A*_*peak*_ ≈ 1) we must increase the metalens aperture ($W$) while also increasing the coherence across the aperture (${\xi }_{0}$).

## Discussion

TCMT is the foundational model and framework for studying infinite, periodic resonant flat optics. In analogy, here we have introduced STCMT as the framework to model space-varying resonant flat optics. Given the importance of TCMT in understanding and modeling Fano resonant metasurfaces, we believe STCMT is a foundational tool for designing nonlocal meta-optics before implementing a concrete geometry. STCMT qualitatively and quantitatively clarifies the classification of nonlocal metasurfaces into two functionalities: wavefront-selective and wavefront-shaping nonlocal metasurfaces. In practice, this allows rapid classification and prototyping of a system to match the requirements of the application. More generally, STCMT enables computation of the idealized performance of a given system, accounting for finite size, space-varying properties, and degrees of freedom manipulatable across the surface. In other words, it sets goalposts (upper bounds on performance) to be reached for in practical implementation under a given set of assumptions (geometry, number of layers, loss, size, reciprocity, etc.).

In addition to its practical advantages, STCMT captures and provides insight into several novel aspect of recent efforts on nonlocal metasurfaces. We clarified that (i) the two hallmarks of a nonlocal phase gradient (shift of band structure in input momentum, and anomalous reflection) are simple consequences of a mixed Fourier transformation in the context of STCMT; (ii) a symmetric response after such mixed Fourier transformation is enforced by reciprocity, revealing that a near-unity efficiency nonlocal phase gradient is a vertically asymmetric phenomenon, in turn revealing the requirement of vertical asymmetry in the structure; and (iii) that spatial selectivity is intrinsically tied to the eigenwave to a degree controlled by the nonlocality length ${\xi }_{0}$ of the q-BIC (which in turn is a quantitative measure of the lateral interaction length, thereby setting the minimum metasurface size required to observe a Fano response, as seen in Fig. 6c).

While we demonstrated the utility of STCMT for understanding and studying diffractive nonlocal metasurfaces, this represents a unified framework for metasurfaces *with or without* the assumption of locality. For instance, the local assumption is recovered if only the main diagonal of the nonlocal matrices is populated, and the effects of nearest neighbors are included when more elements than just the main diagonal are nonzero. As discussed further in Supplementary Section S15, our framework may enable future work explicitly taking into account neighbor-neighbor (nonlocal) interactions. Additionally, as showcased in Fig. 6, our framework may include both local and nonlocal metasurface behaviors simultaneously, as required for thermal emission control^35^. Future directions may extend our results to study the resonant dynamics of several spectrally overlapping nonlocal modes^6^, leaky-wave metasurfaces based on traveling wave q-BICs^47^, the effects due to partial coherence^48^, and even nonreciprocal behavior^49^. Finally, Supplementary Section S16 discusses the limitations of the present study. STCMT elegantly captures the essential physics of these systems, and enables rapid, analytical and numerical study and design of next-generational meta-optical system. These emerging platforms are capable of unprecedent control of light for applications such as augmented reality, secure optical communications, compact optical sources, and nonlinear and quantum optics.

## Materials and methods

The calculations for Figs. 3–6 were carried out using the matrix form of STCMT, described in the supplementary information. The calculations for Fig. 2 were carried out using the analytical forms of TCMT and STCMT, described in the supplementary information. The full-wave simulations were carried out using Lumerical FDTD solutions. Example codes of the matrix form of STCMT are provided in the supplementary information.

### Supplementary information

Supplementary Materials

## Data Availability

All data needed to evaluate the conclusions in this study are presented in the paper and in the supplementary materials.
