# Supplementary material for: Spatio-temporal coupled mode theory for nonlocal metasurfaces
Source: Light Sci Appl. 2024 Jan 24;13:28. doi: 10.1038/s41377-023-01350-9 (PMC11251021; doi:10.1038/s41377-023-01350-9)

Supplementary Materials for:
Spatio-temporal coupled mode theory for nonlocal metasurfaces

Adam Overvig[1], Sander Mann[1], and Andrea Alù[1,2,*]

[1]Photonics Initiative, Advanced Science Research Center, City University of New York, New York, NY 10031, USA
[2]Physics Program, Graduate Center of the City University of New York, New York, NY 10016, USA
*Corresponding author: aalu@gc.cuny.edu

Contents

S1. Temporal Coupled mode theory 2
- **S1a. General development of TCMT** 2
- **S1b. TCMT for the device class of interest** 4
- **S1c. Circular polarization** 12

S2. Obtaining the STCMT dynamical equations 13
- **S2a. Using a Taylor Expansion** 13
- **S2b. Using a cosine series expansion** 15

S3. Propagator and Green's Function 16

S4. Comparison to bare interface 17

S5. Physical constraints 18
- **S5a. Equation (60)** 19
- **S5b. Equation (61)** 21
- **S5c. Equation (62)** 22

S6. Space-frequency description of aperiodic nonlocal metasurfaces 23

S7. Phase gradient on and off the band edge frequency 25

S8. Matrix form on a discrete grid 31

S9. Boundary conditions for finite and infinite metasurfaces 34

S10. 2D nonlocal metasurfaces 38

S11. Nonlocal metalenses off the band edge frequency 40

S12. Wavefront-shaping vs wavefront-selective regimes 42

S13. Spatial selectivity and eigen-waves 43

S14. Thermal metasurfaces off the band-edge frequency 45

S15. Applicability to local and nonlocal metasurface design 46

S16. Limitations of the present study 47

References 48

S17. Example MATLAB code, reproducing Fig. 5b of the maintext 50

S18. Example MATLAB code, reproducing thermal metalens from Fig. 6 52

S1. Temporal Coupled mode theory

S1a. General development of TCMT

Periodic nonlocal metasurfaces are often studied for their resonant properties, which are well-captured by temporal coupled mode theory (TCMT). Here, we review the fundamentals of TCMT and compare the numerical model with full-wave simulations of a conventional, periodic q-BIC device. In this section, the TCMT begins by equations of motion in the time-domain, assumes time-harmonic solutions to yield equations in the frequency domain, and then Taylor expands the resonant frequency as a function of momentum to yield scattering coefficients in the momentum-frequency domain. The main results of the paper are achieved by extending this approach to the space-frequency domain using suitable Fourier transformation and spatial adjustment of the q-BIC coupling properties.

We begin with the equation of motion for a q-BIC with complex modal amplitude $a(t)$ excited by an incoming wave $|s_+\rangle$, where $|a(t)|^2$ is normalized to be the energy stored in the q-BIC per unit area at time t and $\langle s_+|s_+\rangle$ the incident intensity:

$$\frac{da(t)}{dt} = -(i\omega_r + \gamma_r + \gamma_i)a(t) + \langle \kappa^* | s_+ \rangle \ . \tag{1}$$

The coupling vector $|\kappa\rangle$ contains the coupling coefficients from free-space excitation, ω_r is the (resonant) modal frequency of the q-BIC, γ_r is the radiative scattering rate, and γ_i is the nonradiative scattering rate (which we will take to be 0 except when discussing Thermal Metasurfaces). We note that the scattering rates may be used interchangably with a

corresponding lifetime, e.g. $\tau_r = 1/\gamma_r$ is the radiative lifetime of the mode. We also wish to know the outgoing (scattered) waves $|s_-\rangle$, which are also normalized so that $\langle s_-|s_-\rangle$ is the outgoing intensity. We assume time-harmonic solutions of the form

$$\begin{aligned} a(t) &= a_0 \exp(-i\omega t) \\ |s_+\rangle &= \exp(-i\omega t)|s_{0+}\rangle, \\ |s_-\rangle &= \exp(-i\omega t)|s_{0-}\rangle \end{aligned} \tag{2}$$

yielding

$$\langle \kappa^* | s_{0+}\rangle = [i(\omega_r - \omega) + \gamma_r] a_0 \tag{3}$$

Then, if in the absence of the q-BIC the outgoing wave is given by

$$|s_{0-}\rangle = C(\omega)|s_{0+}\rangle, \tag{4}$$

where $C(\omega)$ is the background scattering matrix, the contribution from the decaying q-BIC means $|s_-\rangle$ must be given by

$$|s_{0-}\rangle = C(\omega)|s_{0+}\rangle + |d\rangle a_0 . \tag{5}$$

Equations (3) and (5) form the general basis of the TCMT in the frequency domain [1],[2]. A more specific TCMT is then developed by (i) applying relevant physical constraints and (ii) parameterizing the system of interest with phenomenological parameters. In doing so, we will retrieve the scattering matrix S relating the incoming waves to the the outgoing waves,

$$|s_{0-}\rangle = S|s_{0+}\rangle, \tag{6}$$

parameterized by quantities rationally controlled through the symmetry properties of q-BICs. Regarding (i), here we are interested in physical systems subject to the constraints of reciprocity, energy conservation, and time-reversal [2]. Reciprocity demands that the coupling into the q-BIC must be equivalent to coupling out, namely,

$$|\kappa\rangle = |d\rangle. \tag{7}$$

Energy conservation means that the intensity coupling out of the q-BIC is constrained by the radiative decay rate, in particular,

$$\langle d|d\rangle = 2\gamma_r. \tag{8}$$

Finally, for time reversal invariant systems, the equations describing a decaying q-BIC with no driving (incoming) waves must, under time reversal, match the equations describing a q-BIC being populated at the radiative decay rate without scattering to any outgoing waves, which requires

$$C|d^*\rangle = -|d\rangle. \tag{9}$$

We may now eliminate the modal amplitude in Eqns. (3) and (5), and then apply the reciprocity condition in Eqn. (7) to obtain the scattering matrix

$$S = C + \frac{|d\rangle\langle d^*|}{i(\omega_r - \omega) + \gamma_r}. \tag{10}$$

S1b. TCMT for the device class of interest

We are interested in particular in the local scattering matrix of the form:

$$C = e^{i\Phi_c} \begin{bmatrix} r_0 & 0 & -it_0 & 0 \\ 0 & r_0 & 0 & -it_0 \\ -it_0 & 0 & r_0 & 0 \\ 0 & -it_0 & 0 & r_0 \end{bmatrix}, \qquad (11)$$

where r_0 and t_0 are real-valued Fresnel coefficients (e.g., as approximated by a thin film interference model). This scattering matrix uses a basis for the fields of the form:

$$E = \begin{bmatrix} E_{1,x} \\ E_{1,y} \\ E_{2,x} \\ E_{2,y} \end{bmatrix}, \qquad (12)$$

where $E_{i,p}$ is the electric field on side i with polarization p. Then we seek the complex coupling coefficients

$$|d\rangle = \begin{bmatrix} d_1 \\ d_2 \\ d_3 \\ d_4 \end{bmatrix} e^{i\Phi_c/2} \qquad (13)$$

where we include the background scattering phase factor for convenience, allowing us to write Eqn. (10) as

$$S = e^{i\Phi_c} \left\{ \begin{bmatrix} r_0 & 0 & -it_0 & 0 \\ 0 & r_0 & 0 & -it_0 \\ -it_0 & 0 & r_0 & 0 \\ 0 & -it_0 & 0 & r_0 \end{bmatrix} + \frac{1}{i(\omega_r - \omega) + \gamma_r} \begin{bmatrix} d_1 d_1 & d_1 d_2 & d_1 d_3 & d_1 d_4 \\ d_2 d_1 & d_2 d_2 & d_2 d_3 & d_2 d_4 \\ d_3 d_1 & d_3 d_2 & d_3 d_3 & d_3 d_4 \\ d_4 d_1 & d_4 d_2 & d_4 d_3 & d_4 d_4 \end{bmatrix} \right\} \qquad (14)$$

The development of the TCMT now proceeds by (1) writing down a phenomenological form of $|d\rangle$ relevant to the geometry in Fig. S1 and then (2) applying the

constraints of conservation of energy, coordinate system invariance, and time-reversal symmetry to determine the complex amplitudes in $|d\rangle$. We begin with step (1) guided by the selection rules. Seen in Fig. S1(a), we study the q-BIC system from Refs. [3]-[5], wherein a symmetric photonic crystal slab, sandwiched between an identical superstrate and substrate, is perturbed distinctly at the top and bottom interfaces. In the absence of this perturbation, the system is two-port symmetric and the q-BIC is fully bound. The presence of a perturbation breaks this symmetry, meaning that scattering to free-space is introduced in such a way that the scattering to sides $p=1$ and $p=2$ (bottom and top, respectively) distinct, in principle. This perturbation is characterized by four parameters: the magnitude of the perturbation at the bottom and top interfaces are δ_1, and δ_2, and specify how deviated the ellipses are from a perfect circle; the orientation angles of the ellipses are characterizes by angles α_1 and α_2 for the bottom and top interfaces while the second ellipse in each layer is oriented at an angle $\alpha_1+90°$ and $\alpha_2+90°$, respectively. As shown in Fig. S1(d), we describe this system as follows: the perturbation at the bottom interface scatters light to polarization ϕ with a strength proportional to the magnitude of the perturbation, δ_1; the perturbation at the top interface scatters light to polarization θ with a strength proportional to the magnitude of that perturbation, δ_2. As shown in Ref. [3],[4], the polarization angles follow closely the relations

$$\begin{aligned} \phi &\approx 2\alpha_1 \\ \theta &\approx 2\alpha_2 \end{aligned}, \tag{15}$$

and, without loss of generality, we may parameterize the perturbation strengths as

$$\delta_1 = \delta_0 \cos(\delta)$$
$$\delta_2 = \delta_0 \sin(\delta) \quad (16)$$

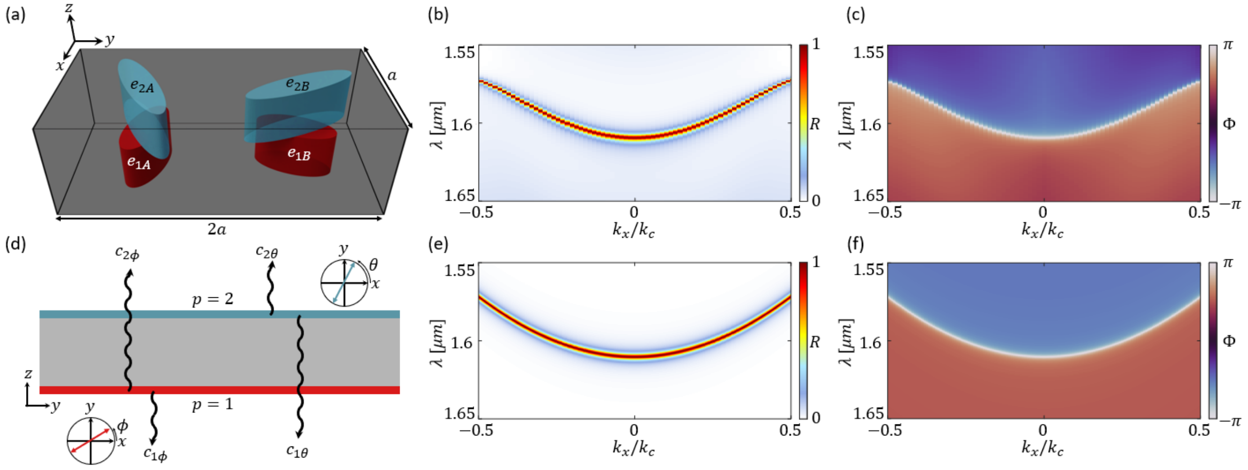

Figure S1. Comparison of a nonlocal metasurface implementation studied by full-wave simulations (a-c) and modeled by TCMT (d-f). (a) Unit cell of a nonlocal metasurface constructed from four elliptical inclusions. (b,c) Reflection and reflected phase for the case in (a) where the ellipses are aligned in the. (d) Schematic of the TCMT model of the device in (a). (e,f) Reflection and reflected phase from a TCMT fit to match the device in (a-c) near $k_x = 0$.

where δ_0 is a measure of the overall perturbation strength and δ is an angular parameter determining the relative contribution from each interface. By construction (or definition for a q-BIC), the Q-factor (or radiative lifetime) is controlled by the overall perturbation strength, and hence we expect $\tau_r \propto 1/\delta_0^2$. And as shown in Ref. [4], δ plays an important role in adjusting the overall phase of $|d\rangle$, motivating this parameterization.

With these assumptions, we may write the components of $|d\rangle$ based on the sum of the scattering events from each interface:

$$
\begin{aligned}
d_1 &= c_{1\phi}\cos(\phi) + c_{1\theta}\cos(\theta) \\
d_2 &= c_{1\phi}\sin(\phi) + c_{1\theta}\sin(\theta) \\
d_3 &= c_{2\phi}\cos(\phi) + c_{2\theta}\cos(\theta) \\
d_4 &= c_{2\phi}\sin(\phi) + c_{2\theta}\sin(\theta)
\end{aligned}
\tag{17}
$$

where the coefficients c_{ip} represent the coupling to port i from the scatterer producing polarization p. These coefficients are not completely independent, per the symmetry of the device. In particular, we may write them in the form

$$
\begin{aligned}
c_{1\phi} &= d_0\cos(\delta)e^{i\Phi} \\
c_{2\theta} &= d_0\sin(\delta)e^{i\Phi} \\
c_{2\phi} &= c_{1\phi}ge^{i\Delta\Phi} \\
c_{1\theta} &= c_{2\theta}ge^{i\Delta\Phi}
\end{aligned}
, \tag{18}
$$

where the first two coefficients represent scattering directly from an interface to their respective ports and the latter two represent indirect scattering from an interface through the slab and to the opposite port. The direct scattering coefficients are not equal in magnitude because the perturbation strengths are different (parameterized by δ), but by the symmetry of the unperturbed system (and thereby, the mode profile), a symmetric mode will scatter with the same direct phase Φ. An anti-symmetric mode will scatter with opposite phase, which may accounted for with δ. The indirect scattering coefficients [the latter two in Eqn. (18)] may be written without loss of generality in terms of their direct counterparts and a relative amplitude g and phase factor $\Delta\Phi$. In our case, the phase factor is $\Delta\Phi = -\pi/2$, following the phase relation of reflected (direct) and transmitted (indirect) light in Eqn. (11) for the vertically symmetry (unperturbed) structure.

We now begin step (2) of developing the TCMT by applying physical constraints by applying the consequence of energy conservation,

$$\langle d | d \rangle = 2\gamma_r \tag{19}$$

and time-reversal symmetry,

$$C | d^* \rangle = - | d \rangle , \tag{20}$$

to determine the allowed forms the complex coefficients. From Eqn. (19) we have

$$|d_1|^2 + |d_2|^2 + |d_3|^2 + |d_4|^2 = 2\gamma_r \tag{21}$$

which, upon inserting Eqn. (17) and Eqn. (18), leads to

$$|d_0|^2 = \frac{2\gamma_r}{1+g^2} . \tag{22}$$

Meanwhile, from time reversal symmetry [Eqn. (20)], we have the constraint

$$r_0 d_1^* - i t_0 d_3^* = -d_1 . \tag{23}$$

from which we have

$$\frac{r_0\left(\cos(\phi)\cos(\delta) + i\cos(\theta)\sin(\delta)g\right) - it_0\left(i\cos(\phi)\cos(\delta)g + \cos(\theta)\sin(\delta)\right)}{\cos(\phi)\cos(\delta) - i\cos(\theta)\sin(\delta)g} = -e^{2i\Phi} . \tag{24}$$

Calling the left-hand side h_0, the right-hand side requires that $|h_0| = 1$, from which it may be shown that

$$g = \frac{1-|r_0|}{\sqrt{1-r_0^{\;2}}}. \tag{25}$$

To simplify the final form, we now note that for the system at study here, we operate such that the resonant frequency ω_r is near the transmission peak of a Fabry-Perot background, $r_0 = 0$, yielding

$$\begin{aligned} &g = 1 \\ &h_0 = 1 \\ &e^{i\Phi} = i \\ &|d_0| = \sqrt{\gamma_r} \end{aligned} \tag{26}$$

And thereby, we arrive at the following form for the scattering coefficients,

$$\begin{aligned} d_1 &= i\sqrt{\gamma_r}\left[\cos(\phi)\cos(\delta) - i\cos(\theta)\sin(\delta)\right] \\ d_2 &= i\sqrt{\gamma_r}\left[\sin(\phi)\cos(\delta) - i\sin(\theta)\sin(\delta)\right] \\ d_3 &= i\sqrt{\gamma_r}\left[-i\cos(\phi)\cos(\delta) + \cos(\theta)\sin(\delta)\right] \\ d_4 &= i\sqrt{\gamma_r}\left[-i\sin(\phi)\cos(\delta) + \sin(\theta)\sin(\delta)\right] \end{aligned} \tag{27}$$

Equation (27) may be written in a more suggestive form by separating the upwards and downward scattering coefficients,

$$\begin{aligned} d_{up} &= \begin{bmatrix} d_1 \\ d_2 \end{bmatrix} = i\sqrt{\gamma_r}\left\{\cos(\delta)\begin{bmatrix}\cos(\phi) \\ \sin(\phi)\end{bmatrix} - i\sin(\delta)\begin{bmatrix}\cos(\theta) \\ \sin(\theta)\end{bmatrix}\right\} \\ d_{down} &= \begin{bmatrix} d_3 \\ d_4 \end{bmatrix} = i\sqrt{\gamma_r}\left\{-i\cos(\delta)\begin{bmatrix}\cos(\phi) \\ \sin(\phi)\end{bmatrix} + \sin(\delta)\begin{bmatrix}\cos(\theta) \\ \sin(\theta)\end{bmatrix}\right\} \end{aligned}, \tag{28}$$

That is, relative to the factor i, real part of the upward scattered state's Jones vector is given by the linear polarization state ϕ with strength $\cos(\delta)$ while the imaginary part is given by the linear polarization state θ with strength $g\sin(\delta)$.

With Eqn. (27) in hand, the elements of $|d\rangle$ are known, and the overall scattering matrix is uniquely determined by the phenomenological parameters $r,\omega_0,\tau_r,\phi,\theta$. We may write the final scattering matrix in the form:

$$S = e^{i\Phi_c}\left[C + \frac{1}{1-i\Omega\tau_r}D\right], \tag{29}$$

with our case specifically parameterized by

$$\begin{aligned}
&C = -i\begin{bmatrix} 0 & 0 & 1 & 0 \\ 0 & 0 & 0 & 1 \\ 1 & 0 & 0 & 0 \\ 0 & 1 & 0 & 0 \end{bmatrix}, \\
&D = \begin{bmatrix} d_1 \\ d_2 \\ d_3 \\ d_4 \end{bmatrix}\begin{bmatrix} d_1 & d_2 & d_3 & d_4 \end{bmatrix}, \\
&\begin{bmatrix} d_1 \\ d_2 \\ d_3 \\ d_4 \end{bmatrix} = \cos(\delta)\begin{bmatrix} \cos(\phi) \\ \sin(\phi) \\ -i\cos(\phi) \\ -i\sin(\phi) \end{bmatrix} + \sin(\delta)\begin{bmatrix} -i\cos(\theta) \\ -i\sin(\theta) \\ \cos(\theta) \\ \sin(\theta) \end{bmatrix}, \\
&\Omega = \omega - \omega_r, \\
&\Phi_c = \pi,
\end{aligned} \tag{30}$$

where we note that the frequency difference Ω may be adjusted to account for nonradiative loss rate γ_i by the replacement

$$\Omega \to \Omega = \omega - (\omega_0 - i\gamma_i). \tag{31}$$

These phenomenological parameters are each directly controlled by geometric (and material) parameters as

$$\begin{aligned}&\omega_0 = f(\epsilon)\\&\tau_r \propto 1/\delta_0^{\,2}\\&\phi \approx 2\alpha_1\\&\theta \approx 2\alpha_2\end{aligned} \tag{32}$$

where $f(\epsilon)$ represents some function (with no generalized closed form) of the period, permittivity, and other geometrical parameters of the unperturbed PCS.

Finally, we may simply extend this model to the momentum-frequency domain by the replacement $\omega_r \to \omega_r(\mathbf{k})$ and by taking the incoming and outgoing wave basis as planewaves with in-plane momenta $\mathbf{k}$. Here, near normal incidence (i.e., a band edge) the resonant frequency may be approximated parabolically as

$$\omega_r(k) \approx \omega_0 + \frac{b}{2}k^2\,, \tag{33}$$

where ω_0 is the resonant frequency at normal incidence (i.e., the band-edge frequency) and b is the Taylor expansion coefficient describing the curvature of the band near the band edge. Figure S1(b) shows the reflection calculated by the FDTD, and Figure S1(e) shows the reflection using the TCMT model fit to the response of this device. Similarly, the phase upon reflection is shown in Fig. S1(c) for FDTD and Fig. S1(f) for TCMT. This excellent agreement validates the use of the analytical form, and motivates its extension to cover the more interesting spatially varying cases shown in Ref. [2].

S1c. Circular polarization

In the main text, we are principally interested in the case where $\delta = \pi/4$ and $\theta = \phi + \pi/2$. In this case, Eqn. (28) becomes

$$
\begin{aligned}
d_{up} &= \begin{bmatrix} d_1 \\ d_2 \end{bmatrix} = i\sqrt{\gamma_r/2}\left\{\begin{bmatrix} \cos(\phi) \\ \sin(\phi) \end{bmatrix} - i\begin{bmatrix} \cos(\phi+\pi/2) \\ \sin(\phi+\pi/2) \end{bmatrix}\right\} \\
d_{down} &= \begin{bmatrix} d_3 \\ d_4 \end{bmatrix} = i\sqrt{\gamma_r/2}\left\{-i\begin{bmatrix} \cos(\phi) \\ \sin(\phi) \end{bmatrix} + \begin{bmatrix} \cos(\phi+\pi/2) \\ \sin(\phi+\pi/2) \end{bmatrix}\right\}
\end{aligned} \tag{34}
$$

which may be written as

$$
\begin{aligned}
d_{up} &= \begin{bmatrix} d_1 \\ d_2 \end{bmatrix} = i\sqrt{\gamma_r/2}\begin{bmatrix} 1 \\ -i \end{bmatrix}\exp(i\phi) \\
d_{down} &= \begin{bmatrix} d_3 \\ d_4 \end{bmatrix} = i\sqrt{\gamma_r/2}\begin{bmatrix} -i \\ 1 \end{bmatrix}\exp(-i\phi)
\end{aligned} \tag{35}
$$

In a circular polarization basis, we therefore have

$$
\begin{aligned}
d_{up} &= i\sqrt{\gamma_r/2}\begin{bmatrix} 1 \\ 0 \end{bmatrix}\exp(i\phi) \\
d_{down} &= i\sqrt{\gamma_r/2}\begin{bmatrix} -i \\ 0 \end{bmatrix}\exp(-i\phi)
\end{aligned} \tag{36}
$$

as used in the main text.

S2. Obtaining the STCMT dynamical equations

S2a. Using a Taylor Expansion

Well within the purview of TCMT is modelling infinitely periodic structure defined by a band structure in momentum-frequency space. In this case, simple transformation yields:

$$
i[\Omega-\omega]a(\omega)=\langle\kappa^*|s_+(\omega)\rangle \tag{37}
$$

$$\left|s_{-}(\omega)\right\rangle = C(\omega)\left|s_{+}(\omega)\right\rangle + a(\omega)\left|d\right\rangle \tag{38}$$

where we introduce the complex resonant frequency $\Omega = \omega_0 - i\gamma$, and assume that the time dependence of the broadband, background scattering is instantaneous, $C \propto \delta(t-t')$ (see the discussion in Ref. [6]). Next, we explicitly include the momentum dependence

$$i\left[\Omega(k)-\omega\right]a(k,\omega) = \left\langle \kappa^{*}(k)\middle|s_{+}(k,\omega)\right\rangle \tag{39}$$

$$\left|s_{-}(k,\omega)\right\rangle = C(k,\omega)\left|s_{+}(k,\omega)\right\rangle + a(k,\omega)\left|d(k)\right\rangle . \tag{40}$$

To proceed, we assume the Taylor expanded form of the complex resonant frequency:

$$\Omega(k) = \left(\omega_0 + ck + \frac{b}{2}k^2 + \ldots\right) - i\left(\gamma_0 + \gamma_1 k + \frac{\gamma_2}{2}k^2 \ldots\right) \tag{41}$$

With Eqn. (41), Fourier transforming Eqns. (39) and (40) gives

$$\frac{da(x,t)}{dt} + i(\omega_0 - i\gamma)a(x,t) + i(c - i\gamma_1)\frac{da(x,t)}{dx} + i(b - i\gamma_2)\frac{d^2a(x,t)}{dx^2} = \int dx' \left\langle \kappa(x,x')\middle|s_{+}(x')\right\rangle \tag{42}$$

$$\left|s_{-}(x)\right\rangle = \int dx' \left(C(x,x')\left|s_{+}(x')\right\rangle + a(x)\left|d(x,x')\right\rangle\right) \tag{43}$$

On the left-hand side, the spatial derivative terms are obtained from the well-known Fourier transform properties that the nth derivative satisfies

$$\mathbf{F}\left\{\frac{d^n}{dx^n}f(x)\right\} = k^n f(k) \tag{44}$$

where $f(k)=\mathbf{F}\{f(x)\}$ is the Fourier transform of $f(x)$. While on the right-hand side we use the convolution theorem.

S2b. Using a cosine series expansion

Since we know ahead of time that the band structure of a q-BIC in an unperturbed nonlocal metasurface is periodic in momentum space, and is even by reciprocity, another natural choice for expanding the resonant frequency is the cosine series:

$$\Omega(k)=\sum_{n=0}^{\infty}\frac{b_n}{x_n^{2n}}\cos\left(x_n k\right)-i\Gamma, \tag{45}$$

where here we keep the loss constant for simplicity, b_n are coefficients and

$$x_n=n\frac{P}{2\pi} \tag{46}$$

for a lattice periodicity P. Then, after Fourier transformation as in the previous section, we now obtain

$$\frac{da(x,t)}{dt}=-i(-i\Gamma)a(x,t)-\frac{i}{2}\sum_{n=0}^{\infty}b_n\left[a(x-x_n,t)+a(x+x_n,t)\right]+\int dx'\left\langle\kappa(x,x')\middle|s_+(x')\right\rangle \tag{47}$$

Now, keeping only the leading order terms $n=0,1$, we find

$$\frac{da(x,t)}{dt}=-\Gamma a(x,t)-\frac{i}{2}\left\{2b_0a(x,t)+\frac{b_1}{x_1^2}\left[a(x-x_1,t)+a(x+x_1,t)\right]\right\}+\int dx'\left\langle\kappa(x,x')\middle|s_+(x')\right\rangle \tag{48}$$

Noticing that a well-known approximation for a second derivative is

$$\frac{d^2a(x,t)}{dx^2} \approx \frac{a(x-x_1,t)+a(x+x_1,t)-2a(x,t)}{x_1^2}, \tag{49}$$

which becomes an equality in the limit that $x_1 \to \infty$, we re-obtain the non-Hermitian Schrodinger equation used in the main text with the equivalents

$$\begin{aligned} \omega_0 &= b_0 + \frac{b_1}{x_1^2} \\ b &= -\frac{b_1}{2} \end{aligned} \tag{50}$$

Given the Taylor series expansion of the cosine function, this result is well within expectation.

S3. Propagator and Green's Function

In absence of the potential V, we have the equation for a free particle, for which the solution is described by the propagator K and the initial condition $a(x,t=0)=a_0(x)$:

$$a(x,t)=\int K(x,x',t)a_0(x')dx' \tag{51}$$

$$K(x,x',t)=\frac{1}{\sqrt{2\pi ibt}}e^{i(x-x')^2/2bt}. \tag{52}$$

The addition of a spatially constant V adds a phase factor independent of x. Hence, in our case the propagator is

$$K(x,x',t)=\frac{1}{\sqrt{2\pi ibt}}e^{i(x-x')^2/2bt}e^{-i\omega_0 t}e^{-\gamma t}. \tag{53}$$

In addition to solving the homogenous equation, the propagator yields the Green's function G_t for the inhomogeneous equation:

$$G_t\left(x,x',t\right)=\frac{1}{2\gamma}\Theta\left(t\right)K\left(x,x',t\right), \tag{54}$$

where $\Theta\left(t\right)$ is the Heaviside step function. Using these functions, we may apply physical constraints such as conservation of energy (which takes the form of a continuity equation), time-reversal invariance and reciprocity (**Supplementary Section S5**).

S4. Comparison to bare interface

At the band-edge frequency, $\omega=\omega_0$, the nonlocal kernel drops to a factor e^{-1} of its maximum value at a distance equal to

$$\xi_0=\sqrt{b\tau_r}\ . \tag{55}$$

This value, which we call the nonlocality length, is the characteristic distance across which the optical response is correlated for the band-edge mode. In general, if this value is sufficiently small, optical power is well-localized, and the metasurface may be considered local. For comparison, we consider scattering of s-polarized light from free-space to a bare interface of a dielectric with refractive index n, described by the Fresnel coefficient

$$r_s(k)=\frac{\sqrt{k_0^2-k^2}-\sqrt{n^2k_0^2-k^2}}{\sqrt{k_0^2-k^2}+\sqrt{n^2k_0^2-k^2}}\ . \tag{56}$$

The nonlocal reflection kernel may be computed by taking the Fourier Transform of Eqn. (56) (setting r_s to 0 when $|k|>k_0$). For comparison, Fig. S2(a,b) reproduce the Green's

Function of the device from the main text, also shown in Fig. S1. Figures S2(c,d) compare the reflectance at the band-edge and magnitude of the nonlocal reflection kernel at the band to the case of a bare interface with $n = 1.45$ (glass). While there are nonzero correlations even for the bare interface (demonstrating that no optical interface is truly local), it is apparent that the ξ_0 for the bare interface is smaller than the wavelength, while it is larger than the wavelength in the q-BIC case. This suggests a natural definition for nonlocality: a metasurface is nonlocal if its nonlocality length is larger the wavelength in the surrounding materials.

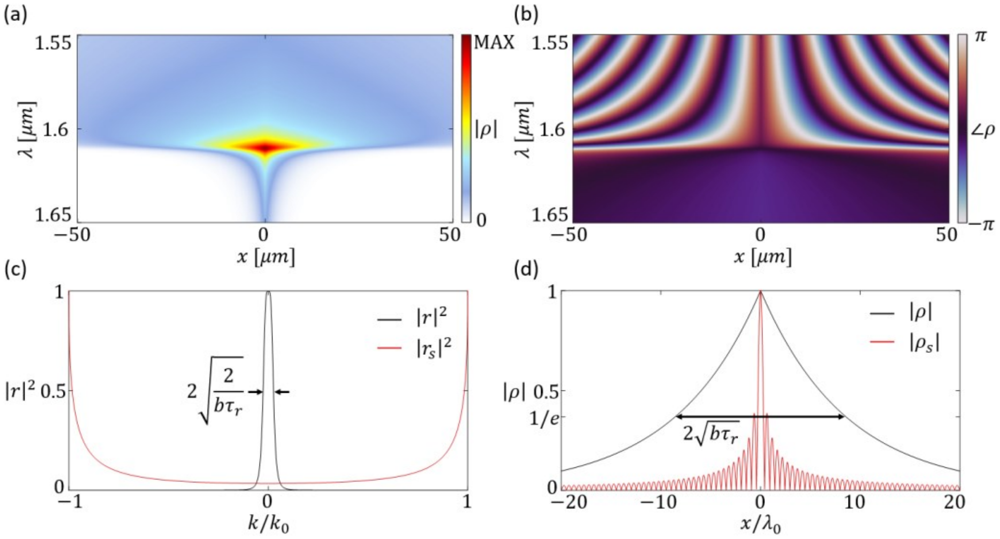

Figure S2. Nonlocal reflection kernel. Amplitude (a) and phase (b) of the nonlocal reflection kernel as a function of x and λ for the device in Fig. S1; copied from the main text Figure 2. (c) Comparison of the reflection coefficient r for the device in Fig. 2 and the Fresnel reflection coefficient r_s for s-polarized light incident on glass. (d) Comparison of the amplitude of the nonlocal kernel for the cases in (c).

S5. Physical constraints

Here we show the derivation that the usual TCMT constraints,

$$\langle d|d\rangle = 2\gamma \tag{57}$$

$$|\kappa\rangle = |d\rangle \tag{58}$$

$$C|d^*\rangle = -|d\rangle \tag{59}$$

hold locally:

$$\langle d(x)|d(x)\rangle = 2\gamma \tag{60}$$

$$|\kappa(x)\rangle = |d(x)\rangle \tag{61}$$

$$C(x)|d^*(x)\rangle = -|d(x)\rangle \tag{62}$$

S5a. Equation (60)

We begin with Eqn. (60), which is derived using conservation of energy. In the case without incoming field, i.e., $|s_+\rangle = 0$, the dynamical equation (9) in the main text becomes the Schrodinger equation (13) in the main text:

$$i\frac{da(x,t)}{dt} = -\frac{b}{2}\frac{d^2a(x,t)}{dx^2} + (\omega_0 - i\gamma)a(x,t) \tag{63}$$

and the out-coupled wave is given by

$$|s_-(x,t)\rangle = a(x,t)|d(x,t)\rangle \tag{64}$$

which carries a scattered power following

$$\left\langle s_{-}(x,t)\middle|s_{-}(x,t)\right\rangle = \left|a(x,t)\right|^{2}\left\langle d(x)\middle|d(x)\right\rangle. \tag{65}$$

Conservation of energy for such a PDE takes the form of a *continuity equation*

$$\frac{d}{dt}\left|a\right|^{2} + \nabla\bullet\mathbf{j} = -\left\langle s_{-}(x,t)\middle|s_{-}(x,t)\right\rangle \tag{66}$$

where the "current" is given by

$$\mathbf{j} = \frac{b}{2i}\left(a^{*}\frac{da}{dx} - a\frac{da^{*}}{dx}\right). \tag{67}$$

where we leave the (x,t) dependence implicit for brevity. Note: this is consistent with conventional quantum mechanics with the replacement $\hbar = 1$ and $b = 1/m$.

By the chain rule, the first time in Eqn. (66) can be written

$$\frac{d}{dt}\left|a\right|^{2} = a^{*}\frac{da}{dt} + a\frac{da^{*}}{dt}. \tag{68}$$

Using Eqn. (63) and its complex conjugate, we obtain

$$\frac{d}{dt}\left|a\right|^{2} = -\frac{ib}{2}a\frac{d^{2}a^{*}}{dx^{2}} + \frac{ib}{2}a^{*}\frac{d^{2}a}{dx^{2}} + 2\gamma\left|a\right|^{2}. \tag{69}$$

Insertion into Eqn. (66) gives

$$2\gamma\left|a(x,t)\right|^{2} = -\left\langle s_{-}(x,t)\middle|s_{-}(x,t)\right\rangle \tag{70}$$

Which, in conjunction with Eqn. (65) finally obtains

$$2\gamma = \langle d(x)|d(x)\rangle. \tag{71}$$

S5b. Equation (61)

Next, we obtain Eqn. (61) using time-reversal invariance and Eqn. (60). We again consider the case without an incident source, but this time consider the time evolution of the mode subject to the initial condition: $a(x,t=0)=a_0(x)$. The modal amplitude at time t is given by the propagator to be

$$a(x,t) = \int dx' a_0(x') K(x,x',t) \tag{72}$$

where

$$K(x,x',t) = \frac{1}{\sqrt{2\pi ibt}} \exp\left[i\frac{(x-x')^2}{2bt}\right] \exp\left[-i\omega_0 t - \gamma t\right], \tag{73}$$

and which decays to an outgoing wave

$$|s_-(x,t)\rangle = |d(x)\rangle \int dx' a_0(x) K(x,x',t). \tag{74}$$

By time-reversal symmetry, the time-reversed excitation of this decay process should re-obtain $a_0^*(x)$ as $t \to 0$. That is, we consider the modal amplitude given excitation by the incoming wave $|s_-^*(x,t)\rangle$ in the dynamical equation (9) of the main text, solved by the Green's Function. In particular, as $t \to 0$ we require

$$a_0^*(x) = \int dx' G_t^*(x,x',t) \langle \kappa^*(x')|s_-^*(x',t)\rangle \tag{75}$$

Inserting Eqn. (74) and writing the Green's Function in terms of the Propagator gives

$$a_0^*(x)=\frac{1}{2\gamma}\int dx' K^*(x,x',t)\left\langle k^*(x')\middle|d^*(x')\right\rangle\int dx'' a_0^*(x'')K^*(x',x'',t) \tag{76}$$

Using the well-known behavior of Eqn. (73) that $K^*(x,x',t)\to\delta(x-x')$ as $t\to 0$, we straightforwardly arrive at

$$a_0^*(x)=\frac{1}{2\gamma}a_0^*(x)\left\langle\kappa^*(x)\middle|d^*(x)\right\rangle \tag{77}$$

Or,

$$2\gamma=\left\langle\kappa^*(x)\middle|d^*(x)\right\rangle \tag{78}$$

Comparison to Eqn. (71) then gives

$$\left|\kappa(x)\right\rangle=\left|d(x)\right\rangle. \tag{79}$$

S5c. Equation (62)

Finally, we derive Eqn. (62). In the same scenario as the previous section, since in the forward-time case there was no source, the time reversed case should also satisfy the condition that no outgoing (scattered) waves are generated as $t\to 0$. That is, as $t\to 0$

$$0=C(x)\left|s_-^*(x,t)\right\rangle+\left|d(x)\right\rangle\int dx' G_t(x,x',t)\left\langle d^*(x')\middle|s_-^*(x,t)\right\rangle \tag{80}$$

Again inserting (74) and rearranging we obtain

$$C(x)\left|d^*(x,t)\right\rangle\int dx' a_0^*(x')K^*(x,x',t)=-\left|d(x)\right\rangle\frac{1}{2\gamma}\int dx' K^*(x,x',t)\left\langle d^*(x')\middle|d^*(x')\right\rangle\int dx'' a_0^*(x'')K^*(x',x'',t) \tag{81}$$

which, as $t\to 0$ and using Eqn. (71) becomes

$$C(x)\left|d^{*}(x)\right\rangle a_0^{*}(x) = -\left|d(x)\right\rangle a_0^{*}(x) \quad (82)$$

or

$$C(x)\left|d^{*}(x)\right\rangle = -\left|d(x)\right\rangle . \quad (83)$$

S6. Space-frequency description of aperiodic nonlocal metasurfaces

Regardless of periodicity, a nonlocal metasurface device may still be studied in the basis of plane waves (especially useful for phase gradient devices). A complete description of the scattering from such a metasurface requires knowledge of its scattering matrix $S(k,k',\omega)$, constructed as

$$S(k,k',\omega) = \begin{bmatrix} S_{11}(k,k',\omega) & S_{12}(k,k',\omega) \\ S_{21}(k,k',\omega) & S_{22}(k,k',\omega) \end{bmatrix}, \quad (84)$$

where k' and k are the basis wavevectors of the incoming and outgoing wavevectors, respectively, and subscripts delineate the two sides of the metasurface interface. For a lossless, reciprocal, system we have by reciprocity,

$$S_{ij}(k_1,k_2,\omega) = S_{ji}(-k_2,-k_1,\omega) \quad (85)$$

and by conservation of energy,

$$\begin{aligned} &\int dk_1 \left[\left|S_{11}(k_1,k_2,\omega)\right|^2 + \left|S_{21}(k_1,k_2,\omega)\right|^2 \right] = 1 \\ &\int dk_1 \left[\left|S_{22}(k_1,k_2,\omega)\right|^2 + \left|S_{12}(k_1,k_2,\omega)\right|^2 \right] = 1 \end{aligned} . \quad (86)$$

In words, in a lossless system the sum of the reflectance and transmittance to all output momenta k_1 must be unity for any input momentum k_2. Note that while in principle k and k' vary continuously from $-nk_0$ to nk_0 for surrounding media of refractive index n, making the dimension of these matrices infinite, in practice discretization will provide sufficient numerical accuracy and will yield a finite scattering matrix in Eqn. (84).

Now we relate the scattering matrix elements to the appropriate nonlocal kernels. By definition, the scattering matrix transforms an incoming field $E_j(k',\omega)$ into an outgoing field $E_i(k,\omega)$,

$$E_i(k,\omega) = S_{ij}(k,k',\omega)E_j(k',\omega), \tag{87}$$

In the space frequency domain, we correspondingly have

$$E_i(x,\omega) = \int dx'\sigma_{ij}(x,x',\omega)E_j(x',\omega). \tag{88}$$

We now seek the relationship between S_{ij} and σ_{ij}. Since we wish to compare this to the scattering matrix elements in a plane wave basis, we use the test incident plane wave

$$E_j(x',\omega) = \exp(ik'x'), \tag{89}$$

in which case Eqn. (88) shows, for instance, the reflected field is simply the Fourier transform of the nonlocal reflection kernel. We may then decompose the reflected field into its constituent plane waves by an inverse Fourier transform

$$E_i(k,\omega) = \int dxE_i(x,\omega)\exp(-ikx) \tag{90}$$

which finally gives the reflection coefficient as

$$S_{ij}(k,k',\omega)=\int dx\int dx'\sigma_{ij}(x,x',\omega)\exp(ik'x')\exp(-ikx). \qquad (91)$$

That is to say, the elements of the scattering matrix may be computed by a mixed Fourier transform of the nonlocal kernels, confirming that the nonlocal kernels fully contain the information required to describe a nonlocal metasurface in the space-frequency domain. Likewise, inverting this relation allows retrieval of the nonlocal kernel given a known scattering matrix.

S7. Phase gradient on and off the band edge frequency

Figure S3 studies four distinct cases: no phase gradient, at the band edge [Fig. S3(a-d)]; $k_G=2\pi/W$, at the band edge [Fig. S3(e-h)]; no phase gradient, off the band edge [Fig. S3(i-l)]; and $k_G=2\pi/W$, off the band edge [Fig. S3(m-p)]. Figure S3(a) shows schematically the nature of the resonant reflection at the band-edge frequency, $\omega=\omega_0$, depicting specular reflection at normal incidence when incident from either side. Figure S3 (b) reports the reflectance from side 1 in this case, showing the parabolic band structure of the q-BIC. Figure S3(c) depicts the amplitude and phase of the nonlocal reflection and transmission matrices from side 1, P and T (note that T is essentially identical to P except along the main diagonal).

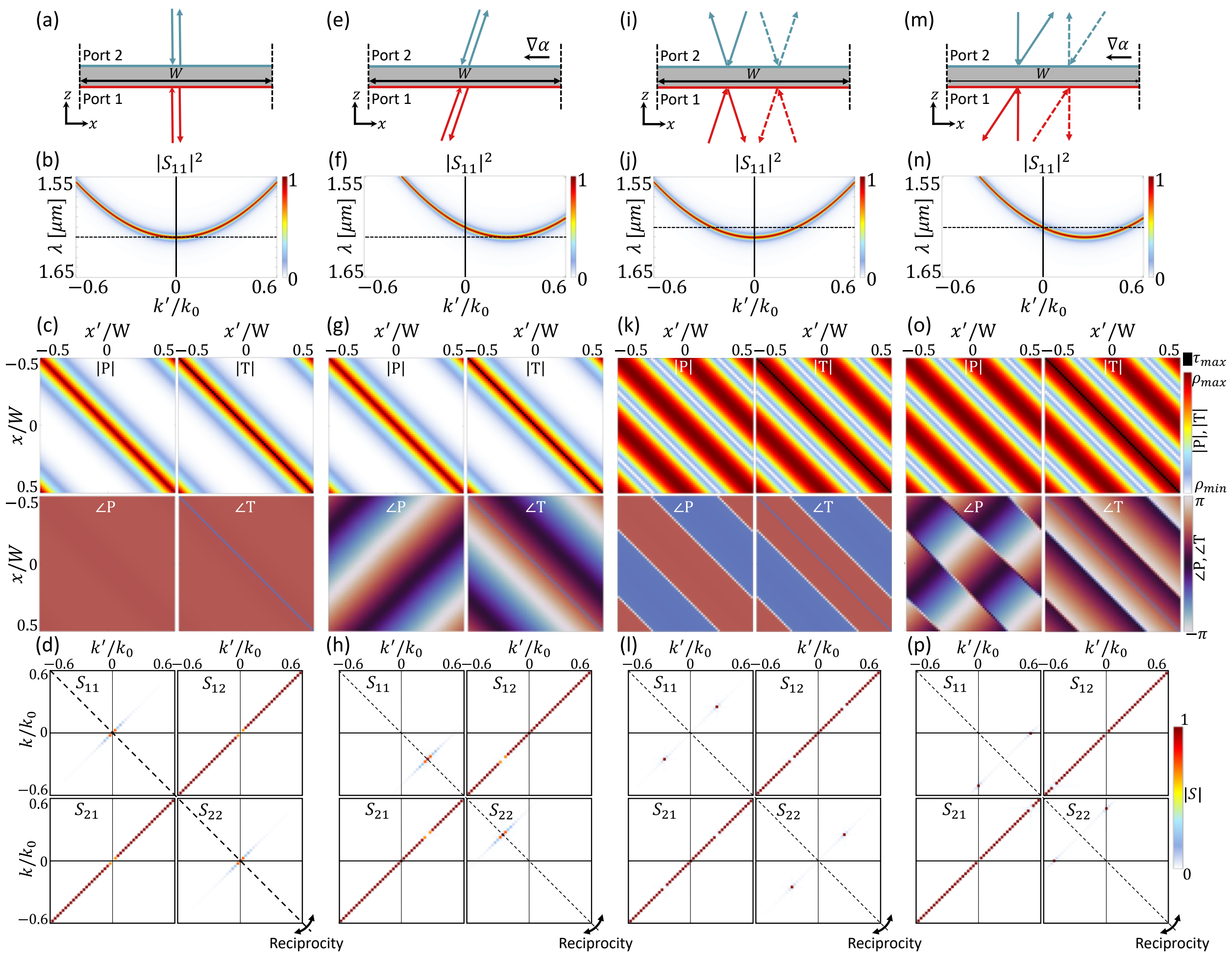

Figure S3. Analysis of phase gradient devices. (a) Schematic of the resonant response of a nonlocal phase gradient device with $k_G = 0$ at the band-edge frequency. (b) Reflectance as a function of incident momentum k' for the device in (a). (c) Nonlocal kernel matrices for the device in (a) at the band-edge frequency. (d) Scattering matrix for the device in (a), showing unity specular reflectance only at normal incidence. (e-h) The same study in (a-d) but for a device with $|k_G| = 2\pi / W$ at the band-edge frequency. (i-l) The same study in (a-d) but at a frequency off the band edge, $\omega_1 = \omega_0 + bk_G^2 / 2$. (m-p) The same study in (a-d) but for a device with $|k_G| = 2\pi / W$ at a frequency off the band edge, $\omega_1 = \omega_0 + bk_G^2 / 2$.

Finally, Fig. S3(d) reports the scattering matrix elements for this case, showing that specular transmission occurs except at normal incident from either side, wherein specular reflection occurs. Note that for sufficient resolution in k-space, Fig. S3(d) is computed using mixed fast Fourier transforms of a version of Fig. S3(c) including 10 periods.

In contrast, with a nonlocal phase gradient the device retroreflects light at the resonant frequency, as shown in Fig. S3(e). From side 1, the band edge mode is shifted in k-space by k_G, meaning that the resonance only occurs for $k' = k_G$. In particular,

$$\omega(k') = \omega_0 + \frac{b}{2}\left(k' - k_G\right)^2 . \tag{92}$$

This shift, as well as the anomalous reflection, is encoded in P and T in the phase information [Fig. S3(g)], and the scattering matrix at the band-edge frequency is depicted in Fig. S3(h), showing that the resonant mode occurs at $k' = k_G$ and $k = -k_G$ when light is incident from side 1, while it occurs at $k' = -k_G$ and $k = k_G$ when light is incident from side 2. Note that this behavior is consistent with the requirements of reciprocity: the scattering matrices must be symmetric about the main diagonal, which corresponds to $k = -k'$, or retroreflection. Hence we see that the action of the phase gradient is to the shift the response along the retroreflection condition by an amount $\left(k_G, -k_G\right)$ (in S_{11}).

Next, we consider the same device in Fig. S3(a-d) but at a frequency $\omega_1 = \omega_0 + bk_G^2/2$, which is the resonant frequency at normal incidence for the phase gradient case [see Eqn. (92)]. In this case, there are two resonant modes existing at $k' = \pm k_G$. As depicted in Fig. S3(i), when $k' = k_G$, light reflects to $k = k_G$, and under time-reversal [dashed arrows in Fig. S3(i)] we thereby naturally have the case that $k' = -k_G$ reflects to $k = -k_G$. The presence of the two modes is captured by the nonlocal kernels in Fig. S3(k), wherein we see the characteristics of a standing wave due to the two counterpropagating modes existing $k' = \pm k_G$. We also note that because we are operating off the band-edge (where Bragg

scattering is maximal) the locality of the mode is substantially reduced. Upon mixed fast Fourier transformation [Fig. 4(l)], the scattering matrices confirm that these two modes correspond to two specular reflections, $k = k' = \pm k_G$, identical when light is incident from both sides (consistent with vertical symmetry).

In contrast, at the same frequency the phase gradient anomalously reflects light at normal incidence to some off-normal angle [Fig. S3(m)]. Naturally, under time-reversal this must correspond to a resonant reflection coming from the anomalously reflected angle and reflecting back to normal incidence [dashed lines in Fig. S3(m)]. As in the case without the phase gradient, these dynamics are due to the presence of two counter-propagating modes. Here, however, instead of being excited at $k' = \pm k_G$, the phase gradient means they are excited at $k' = k_G \pm k_G$ from side 1 and $k' = -k_G \pm k_G$ from side 2. Again, these interesting dynamics are encoded in the nonlocal kernels [Fig. S3(o)], where the characteristics of counter-propagating modes are apparent but modified by a Bloch wave vector. The resulting scattering matrices confirm the picture in Fig. S3(m): the anomalous reflection angle is equal and opposite for normally incident light from side 1 versus side 2, and each of these events have a reciprocal copy (i.e., born of symmetry about the main diagonal) matching the dashed lines in Fig. S3(m).

Finally, we may also use STCMT in conjunction with the angular spectrum method to compute and visualize the scattered fields at example frequencies and incident angles, for a more intuitive view of the scattering behavior portrayed in Fig. S3(h) and (p). Figure S4(b) shows the angular selectivity at the band edge frequency of the nonlocal phase gradient device whose band structure is shown Fig. S4(a). Figure S4(c) depicts both the

reflected and transmitted fields at three example incident angles for the band edge frequency. At the retroreflection condition, $\theta_{in} = \theta_g$, where $\theta_g = \sin^{-1} -k_g/k_0$, we see only (retro)reflected light (middle panel of Fig. S4(c)). But away from this incident angle, we see mostly transmitted fields. As in the case of local metasurfaces, the fields that are reflected follow the Generalized Snell's Law as usual; in contrast to the case of local metasurfaces, here we have an efficiency curve that is highly non-uniform as a function of incident angle (Fig. S4(b)). Similarly, Figs. S4(b,c) explore a frequency off the band edge, in particular, the frequency at which the shifted band structure intersects normal incidence. Here, in contrast, we see two peaks in the reflectance as a function of incident angle (Fig. S4(d)). The first corresponds to normally incident light being anomalously reflected near $-2\theta_g$ (left panel of Fig. S4(e)); the second corresponds to the reciprocal counterpart, namely light incident at $2\theta_g$ being reflected to the device normal (Fig. S4(e)). Meanwhile, the light is inefficiently retroreflected when $\theta_{in} = \theta_g$ (middle panel of Fig. S4(e)). Compared to Fig. S3, the single peak of Fig. S4(b) corresponds to the isolated scattering peak in the S_{11} quadrant of Fig. S3(h). Likewise, the two peaks of Fig S4(d) correspond to the two peaks in the S_{11} quadrant of Fig. S3(p). Note that the phase gradient in Fig. S3 is more selective than that of Fig. S4, so the peaks are broader in Fig. S4.

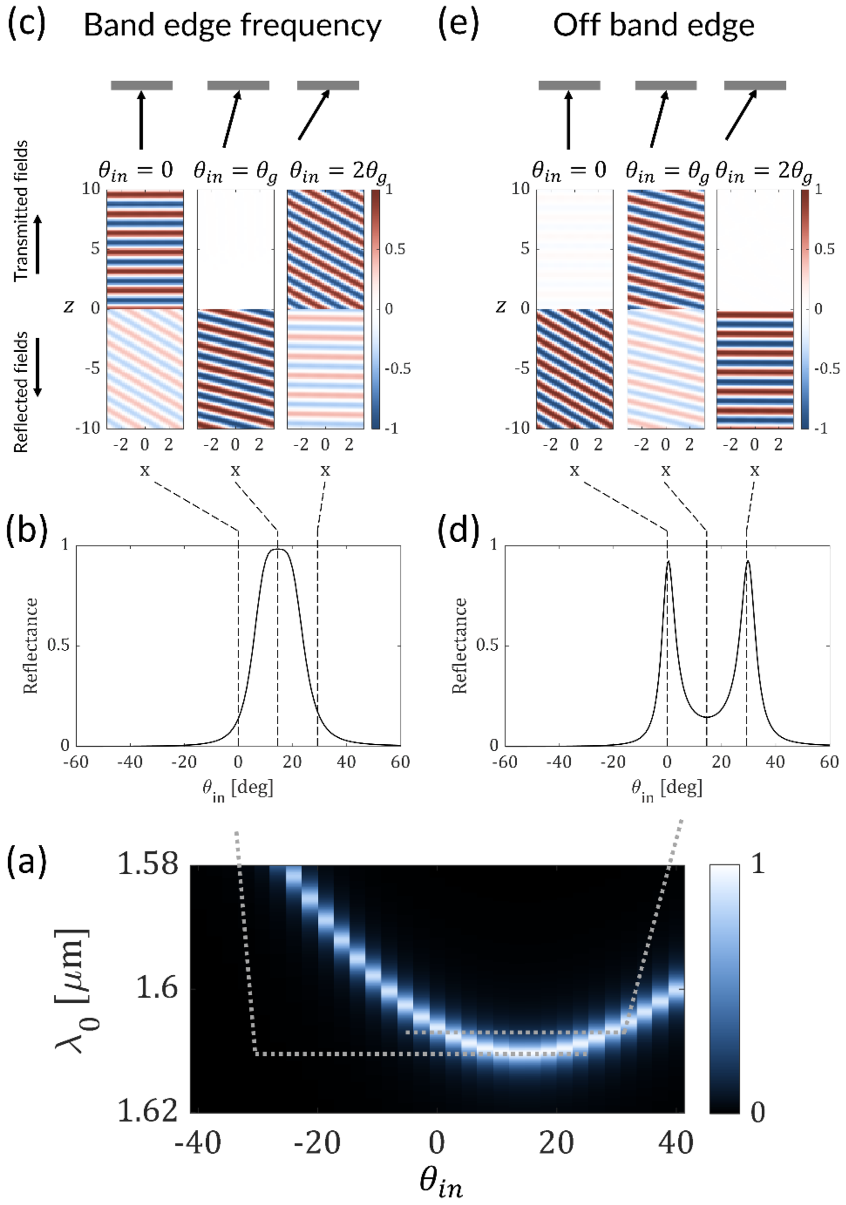

Figure S4. Analysis of a phase gradient device. (a) Reflectance map as a function of incident angle and wavelength (band structure) of a phase gradient device excited from side 1. (b) Reflectance at the band edge frequency, as a function of incident angle. (c) Scattered fields near the center of the phase gradient device for three example incident angles. (d,e) Same as (b,c) but for a frequency off the band edge.

S8. Matrix form on a discrete grid

Next, we show that the continuous form of the STCMT equations may be translated to discrete, or matrix form. This is done for convenience of numerical computation, and is in keeping with the tradition of metasurfaces as being built from discrete subwavelength building blocks (meta-units).

Consider, for instance, the scalar case wherein the modal parameters are invariant. For such a metasurface having meta-units at positions

$$\mathbf{x} = \begin{bmatrix} x_1 & x_2 & \dots & x_n \end{bmatrix}^T , \tag{93}$$

we are interested in the response to incident electric fields of the form

$$\mathbf{E}(\omega) = \begin{bmatrix} E_1(\omega) & E_2(\omega) & \dots & E_n(\omega) \end{bmatrix}^T , \tag{94}$$

where $E_i(\omega) = E(x_i, \omega)$, after interacting with a nonlocal metasurface described by the discrete phase profile

$$\mathbf{\Phi} = \pm \begin{bmatrix} \Phi_1 & \Phi_2 & \dots & \Phi_n \end{bmatrix}^T , \tag{95}$$

where $\Phi_i = \Phi(x_i)$ and the positive (negative) sign applies to light coming from port 1 (2). For instance, in the main text the phase functions followed $\Phi(x) = \pm 2\alpha_1(x)$. Throughout we assume that the n meta-units are equally spaced such that $x_i - x_{i-1} = a$. The reflected and transmitted fields may be calculated by

$$\begin{aligned} \mathbf{E}_r(\omega) &= \mathrm{P}(\omega)\mathbf{E}_{in}(\omega) \\ \mathbf{E}_t(\omega) &= \mathrm{T}(\omega)\mathbf{E}_{in}(\omega) \end{aligned} , \tag{96}$$

where

$$\begin{aligned}\mathrm{P}(\omega) &= i\frac{a}{b\tau_r}\xi(\omega)\exp\left(-\frac{|X-X'|}{\xi(\omega)}\right)e^{i\Phi_X}e^{i\Phi_{X'}}\\ \mathrm{T}(\omega) &= -iI+\frac{a}{b\tau_r}\xi(\omega)\exp\left(-\frac{|X-X'|}{\xi(\omega)}\right)e^{-i\Phi_X}e^{i\Phi_{X'}}\end{aligned} \quad (97)$$

are the nonlocal reflection and transmission kernels in matrix form (we note that the matrix forms are denoted by capitalized ρ and τ). Here, I is the $n\times n$ identity matrix, the factor a is included for normalization purposes (the choice to place it here is purely for convenience), and the $n\times n$ position matrices are given by

$$\begin{aligned}X &= [\mathbf{x} \quad \mathbf{x} \quad \dots \quad \mathbf{x}]\\ X' &= X^T\end{aligned}, \quad (98)$$

where X has n copies of $\mathbf{x}$. Hence, along a row the input position x' varies and along a column the output position x varies. Last, the phase matrices Φ_X and $\Phi_{X'}$ are populated in the same fashion as Eqn. (98):

$$\begin{aligned}\Phi_X &= [\mathbf{\Phi} \quad \mathbf{\Phi} \quad \dots \quad \mathbf{\Phi}]\\ \Phi_{X'} &= \Phi_X^T\end{aligned}. \quad (99).$$

Then, the output fields due to an excitation $\mathbf{E}_{in}$ are obtained by

$$\begin{aligned}\mathbf{E}_r(\omega) &= \mathrm{P}(\omega)\mathbf{E}_{in}(\omega)\\ \mathbf{E}_t(\omega) &= \mathrm{T}(\omega)\mathbf{E}_{in}(\omega)\end{aligned} \quad (100)$$

while the reflectance and transmittance are then calculated simply as

$$R(\omega) = \frac{\left\| \mathrm{P}(\omega)\mathbf{E}_{in}(\omega) \right\|^2}{\left\| \mathbf{E}_{in}(\omega) \right\|^2} ,$$
$$T(\omega) = \frac{\left\| \mathrm{T}(\omega)\mathbf{E}_{in}(\omega) \right\|^2}{\left\| \mathbf{E}_{in}(\omega) \right\|^2} \tag{101}$$

where $\left\| \mathbf{E} \right\|$ is the norm of the vector $\mathbf{E}$.

Using the matrix form, we may also determine the eigen-wave of a discrete nonlocal metasurface by the eigenvector with maximal eigenvalue from the eigenvalue problem

$$R_{eig}\mathbf{E}_{eig}(\omega) = \mathrm{P}^{\dagger}(\omega)\mathrm{P}(\omega)\mathbf{E}_{eig}(\omega) , \tag{102}$$

where $\dagger$ refers to the Hermitian conjugate. In full there will be n eigenvalues and eigenvectors; however, we are primarily interested in the eigenvector with the highest eigenvalue, which is the principal eigen-wave.

Finally, we note that the discretization introduces artificial spatial frequencies of magnitudes $m2\pi / a$, where m is an integer. This numerical approximation produces artificial resonances at frequencies satisfying

$$\omega = \omega_0 + \frac{b}{2}\left(m\frac{2\pi}{a} \right)^2 . \tag{103}$$

Therefore, the period a should be sufficiently small so as to shift these spurious modes out of the frequency range $\Delta\omega$ of interest, which will be the case if

$$a < 2\pi\sqrt{\frac{|b|}{2\Delta\omega}} . \tag{104}$$

Note the dependence on b: as the mode becomes more localized, a finer discretization is naturally required. The period a must also be small enough to sufficiently sample the maximum phase gradient of the device, as usual for metasurfaces:

$$a < \frac{1}{N_p}\frac{\partial \Phi}{\partial x} = \frac{1}{N_p}\frac{2\pi}{P_0}, \tag{105}$$

where $N_p \geq 3$ is the desired number of phase points to be sampled as phase evolves across 2π within a distance P_0.

S9. Boundary conditions for finite and infinite metasurfaces

The results of the previous section are in practice incomplete: we must take care to specify the boundary conditions. We are interested here in two boundary conditions: (i) radiative boundaries and (ii) periodic (Bloch) boundaries. We will consider each in turn. (We note that reflective boundaries may also be of interest, for instance to model guided mode resonance gratings placed between distributed Bragg reflectors [5].)

In case (i), we wish to study devices of finite size. For a finite metasurface, the energy in a q-BIC at position x' is not correlated with positions x that exist outside the metasurface. Hence the value of ρ or τ must vanish accordingly. Happily, this boundary condition happens naturally following the procedure of the previous section. For instance, we consider the matrix form P at the band-edge frequency for the device in Fig. S3 but having a finite width $W = 100\mu m$. As depicted in Fig. S5(a), this device is truncated in-plane, having radiative boundaries. The absolute value of P is depicted in Fig. S5(b) [while the argument of P is shown in Fig. S5(c)], showing peak correlation along the main diagonal and

dropping exponentially off the main diagonal. Note that the main diagonal are the only populated entries in an ideally local device (see the identity factor in T). At the upper left and lower right corners of the matrix we can see the boundary condition: locations outside the matrix implicitly encode a zero value. Considered another way, we could extend the matrix by including values of x and x' outside the depicted range, and populate the matrix along the diagonal according to the local reflection (which is 0). This 'zero-padded' version would naturally be identical. Finally, Fig. S5(d) depicts the principle eigen-wave for this device, normalized to its peak value. While in the infinite case we expect a planewave, in the finite case we see that the eigen-wave's amplitude drops towards the boundaries, apparently minimizing the radiative losses. Still, the eigen-wave has a non-zero value near the boundaries, and this non-ideality results in the reflectance not being unity: the eigenvalue in this case is $R_{eig} = 0.977$. Naturally, as W increase the eigen-wave better approximates a planewave, and R_{eig} approaches unity.

In case (ii), we wish to capture infinite devices, in which case we should expect the deviations from a plane wave should vanish. In the periodic case we require that the energy be correlated according to contributions from every period of the device. For instance, the left edge of the period (near $-W/2$) must be closely correlated to the right edge of the period (near $W/2$), which is not captured in the above procedure or the matrix in Fig. S5(b). Instead, we must alter the construction of P in order to include contributions from every period. For instance, for normally incident light, we sum instances of the nonlocal term shifted by integer multiples of the period W of the device:

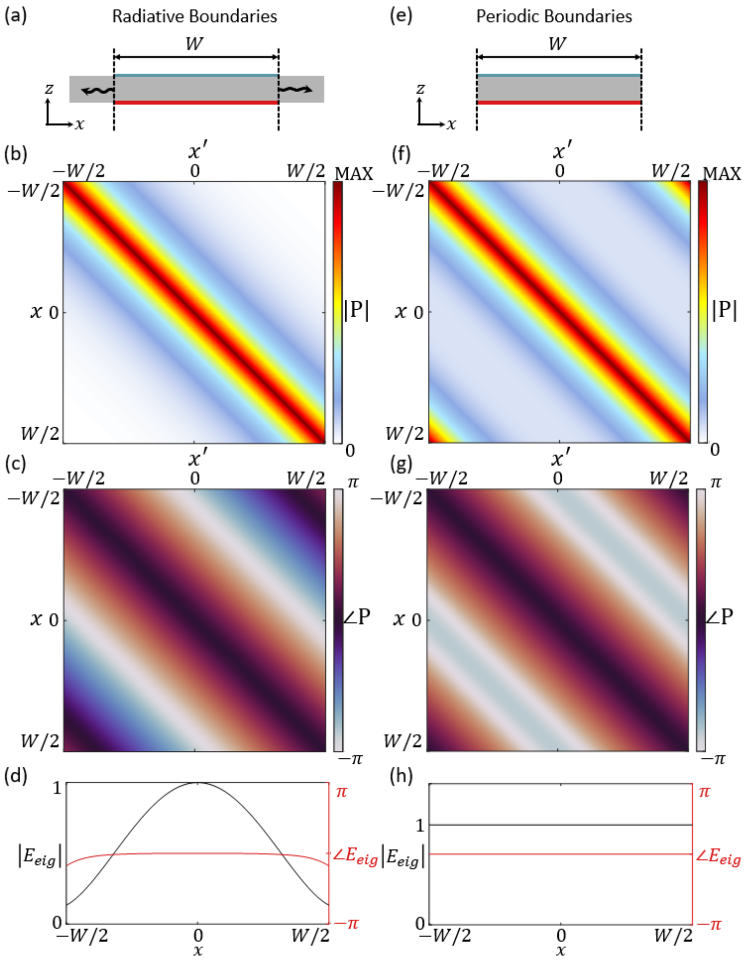

Figure S5. Boundary conditions for finite nonlocal kernel matrices. (a) Schematic showing radiative boundaries, wherein power in the q-BIC is not correlated with positions outside the metasurface width $W = 50\mu m$. (b,c) Amplitude and phase of the nonlocal kernel matrix P with radiative boundaries for a finite version of the device in Fig. 2. (d) Principle eigen-wave for the device in (b,c). (e) Schematic showing periodic boundaries, wherein power in the q-BIC is correlated periodically with period W. (f,g) Amplitude and phase of the nonlocal kernel matrix P for a periodic case of the device in Fig. 2. (h) Principle eigen-wave for the device in (f,g).

$$\begin{aligned} \mathrm{P}(\omega) &= \sum_{j=-N}^{N} i \frac{a}{b\tau_r} \xi(\omega) \exp\left(-\frac{\left|X - X' - jW\right|}{\xi(\omega)}\right) e^{i\Phi_X} e^{i\Phi_{X'}} \\ \mathrm{T}(\omega) &= -iI + \sum_{j=-N}^{N} \frac{a}{b\tau_r} \xi(\omega) \exp\left(-\frac{\left|X - X' - jW\right|}{\xi(\omega)}\right) e^{-i\Phi_X} e^{i\Phi_{X'}} \end{aligned}, \qquad (106)$$

where $2N+1$ is the number of periods accounted for. (Note that $\Phi_X = \Phi_{X-jW}$ by the periodicity of the device.) Naturally, as N approaches infinity the contributions of every period in the infinite device is included, in which case the series converges to give

$$\begin{aligned} \mathrm{P}(\omega) &= i\frac{a}{b\tau_r}\xi(\omega)\operatorname{csch}\left(\frac{W}{2\xi(\omega)}\right)\cosh\left(\frac{W-2\left|X-X'\right|}{2\xi(\omega)}\right)e^{i\Phi_X}e^{i\Phi_{X'}} \\ \mathrm{T}(\omega) &= -iI+\frac{a}{b\tau_r}\xi(\omega)\operatorname{csch}\left(\frac{W}{2\xi(\omega)}\right)\cosh\left(\frac{W-2\left|X-X'\right|}{2\xi(\omega)}\right)e^{-i\Phi_X}e^{i\Phi_{X'}} \end{aligned} \tag{107}$$

Figure S5(e) schematically depicts a periodic device with period $W = 100\mu m$, and the resulting matrix P is shown in Figs. S5(f,g). The boundary conditions are apparent here especially in the upper right and lower left corners of Fig. S5(f), where we see that the main diagonal appears to 'wrap', accounting for the periodicity. Finally, Fig. S5(h) shows the normalized eigen-wave for this case, which is a plane wave with eigenvalue is $R_{eig} = 1$, as expected.

Lastly, since the boundary conditions must be consistent with both the device and the illumination, for light incident with momentum k' we include the Bloch wave in each period of the summation:

$$\begin{aligned} \mathrm{P}(k',\omega) &= i\sum_{j=-N}^{N}\frac{a}{b\tau_r}\xi(\omega)\exp\left(-\frac{\left|X-X'-jW\right|}{\xi(\omega)}\right)e^{i\Phi_X}e^{i\Phi_{X'}}e^{ik'W} \\ \mathrm{T}(k',\omega) &= -iI+\sum_{j=-N}^{N}\frac{a}{b\tau_r}\xi(\omega)\exp\left(-\frac{\left|X-X'-jW\right|}{\xi(\omega)}\right)e^{-i\Phi_X}e^{i\Phi_{X'}}e^{ik'W} \end{aligned} \tag{108}$$

However, the mixed Fourier transform gives the scattering matrices as a function of both k' and k, allowing us to simply use the case without the Bloch wave seen in Eqns. (107).

S10. 2D nonlocal metasurfaces

In this section, we briefly demonstrate the extension of the STCMT to two-dimensional (2D) metasurfaces. This required extending the nonlocal kernels to be a function of both x and y: $\rho(x,x',y,y',\omega)$ and $\tau(x,x',y,y',\omega)$, making the corresponding discrete matrix forms four-dimensional. However, we can flatten the 4D structure into a 2D array of matrices for ease of use and visualization. The 2D array is $N_y \times N_y$ in dimension, where N_y is the number of y positions in the metasurface. Rows of the array correspond to changing y', while columns correspond to changing y. Each element is then a $N_x \times N_x$ matrix as in the above sections, where N_x is the number of x positions.

As an example, Fig. S6(a) depicts the reflection matrix for the case of a radial nonlocal metalens, discretized from

$$\rho(x,x',y,y',\omega)=\frac{i}{b\tau_r}\xi(\omega)\exp\left(-\frac{\sqrt{(x-x')^2+(y-y')^2}}{\xi(\omega)}\right)e^{i\Phi(x,y)}e^{i\Phi(x',y')}, \tag{109}$$

with

$$\Phi(x,y)=-k_0\sqrt{x^2+y^2+f^2}\,. \tag{110}$$

For simplicity, we assume that the band structure is completely isotropic. Figure S6(b) shows the central few elements of the array in Fig. S6(a), overlaid with the indexing of this array described above. Figures S6(c,d) similarly show the phase of the reflection matrix. Each element of the array resembles the cylindrical lenses studied in the previous section, but the radial phase of function of the device is recovered by reshaping the main diagonal

of Fig. S6(c) onto the corresponding (x,y) grid, depicted in Fig. S6(e). As usual, we expect the characteristic response to be encapsulated by the eigen-wave of this device. Figures S6(f,g) depict the amplitude and phase of the metalens, where a similar reshaping is done to the eigenvector for visualization purposes. Finally, Fig. S6(h) depicts the refocused spot at $z=-f$ after excitation by the eigen-wave, consistent with expectation.

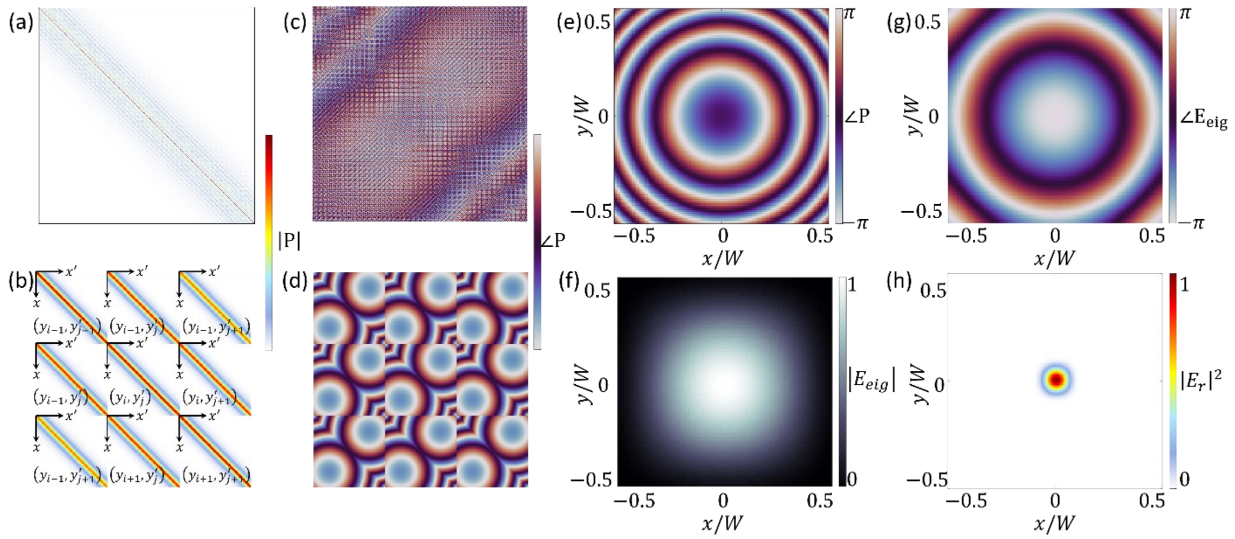

Figure S5. 2D nonlocal metalens. (a,c) Amplitude and phase of the nonlocal reflection kernel, a 2D array of matrices. (b,d) Zoom-in of the central regions of (a,c), overlaid with indexing of the array of matrices. (e) Main diagonal of (c), reshaped onto an (x,y) grid to depict the characteristic phase function of a metalens. (f,g) Amplitude and phase of the computed eigen-wave, which focuses upon reflection at $z=-f_0$. (h) Intensity of the reflected light at the focal plane.

Lastly, we note that the matrix size (which has important consequences for memory requirements and other computational costs) scale as N^4, where N is the characteristic number of positions in each x and y. This compares poorly to full-wave simulations, in which the field matrices scale as N^2 for fixed z dimension simulations. However, it is

apparent from Fig. S6 that the vast majority of elements are 0, suggesting the use of sparse matrices. In this case, we recover the N^2 scaling once the dimension W of the device is larger than the nonlocality length: increasing the number of positions only effectively extends the region near the main diagonal, while all the other elements remain 0.

S11. Nonlocal metalenses off the band edge frequency

Here, we extend our study of the nonlocal metalenses to frequencies off the band edge. We begin by computing the principle eigen-wave at three frequencies and studying how they propagate. Figure S7(a-c) shows the intensity of the reflected eigen-wave at the band edge [Fig. S7(a)], a frequency $\omega_0+\Delta\omega$ [Fig. S7(b)] and $\omega_0+2\Delta\omega$ [Fig. S7(c)], where $\Delta\omega=0.01\mu m^{-1}$. The band-edge eigen-wave refocuses to a point $z=-f$, as expected, but at the two other frequencies, we see that this focal spot as split into two comatic focuses off the optical axis, where the split is larger for the case that is further off the band edge. In parallel, we compute the reflectance as a function of (x_0,z_0) at the same three frequencies, and comparing the results [Figs. S7(d-f)] shows that the strong relationship between the eigen-wave and spatial selectivity extends off the optical axis and off the band edge. We also see that the selectivity increases drastically off the band edge, consistent with the fact that the nonlocality length increases off the band edge (due to reduced Bragg scattering).

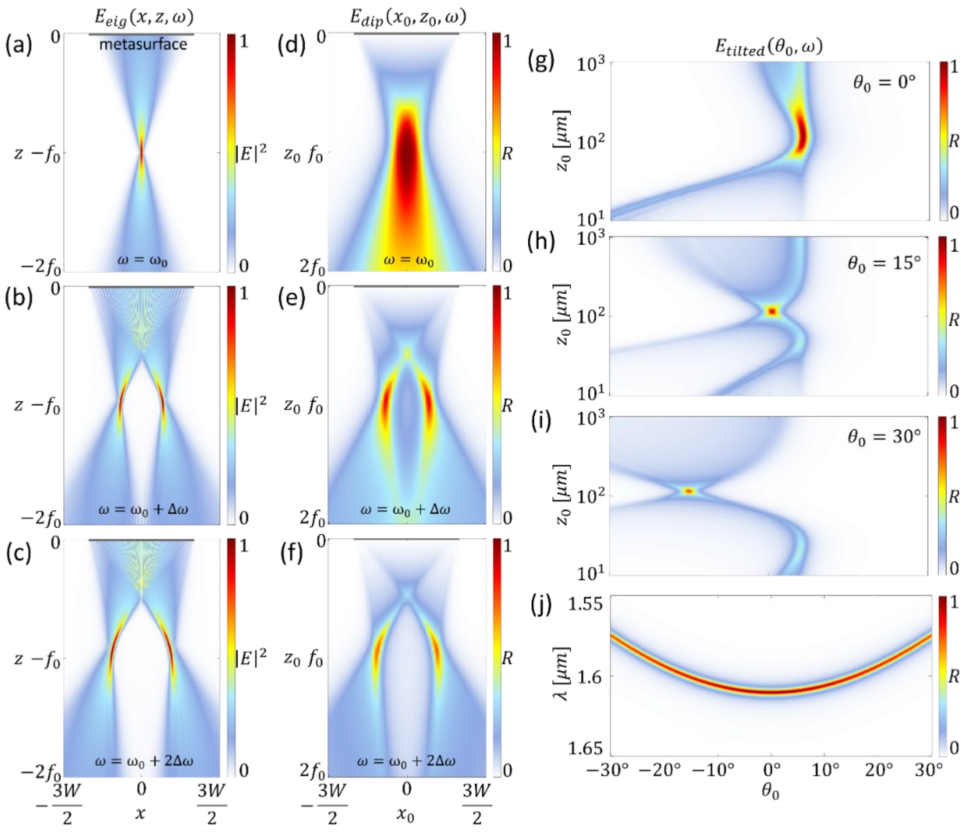

Figure S7. Eigen-waves and spatial selectivity, on and off the band-edge. (a) Eigen-wave for a metalens at $\omega=\omega_0$, propagating downwards after reflection from the metasurface at $z=0$. (b,c) Eigen-waves as in (a) but at frequencies $\omega=\omega_0+\Delta\omega$ and $\omega=\omega_0+2\Delta\omega$, where $\Delta\omega=0.01\mu m^{-1}$. Note the comatic focal responses. (d) Reflectance due to an ideal point source as a function of the position of the source, (x_0,z_0). (e,f) Response as in (d) but at frequencies $\omega=\omega_0+\Delta\omega$ and s $\omega=\omega_0+2\Delta\omega$. (g) Reflectance due to a point source placed along the optical axis ($x_0=0$). (h,i) Reflectance due to a point source placed along the optical axis but for a modification of a tilt factor $\exp\left[ink_0\sin(\theta_0)\right]$ with tilt angles θ_0. (j) Reflectance due to tilted point sources placed at $(0,-f_0)$ as a function of θ_0, recovering the band structure of the q-BIC.

To understand this behavior further, we study the reflectance spectra due to a modified excitation

$$E_{in}(x,\omega)=\exp\left(ik_0\sqrt{x^2+z_0^2}\right)\exp\left[ik_0\sin\left(\theta_0\right)\right], \tag{111}$$

which represents a point source with a momentum shift (e.g., as if it were deflected by a local metasurface before impinging on the nonlocal metasurface). Figures S7(g-j) depict the spectral reflectance for three values of θ_0, showing that the peak reflectance stays at $z_0 = f$ but blue shifts with larger θ_0. This suggests that the modes off the band edge are involved; indeed, Fig. S7(k) depicts the reflectance when $z_0 = f$ as a function of θ_0, recovering the underlying band structure of the q-BIC.

From these results, we see that the q-BIC off the band edge is selective for waves having the characteristics of the eigen-wave at the band edge, but modified by the momentum of the off-band-edge modes. Yet when a single mode is involved, as discussed in the previous section, the reflected wave is not a time-reversed copy. Instead, just as the reciprocal copies of reflection events in the phase gradient case (see Fig. S3), we have two reciprocal focal spots: when a point source excites the nonlocal metalens at one of the foci in Fig. S7(e), for instance, the reflected light refocuses to the other focus. Hence, a superposition of light originating from both foci will refocus light back to both foci; this is the eigen-wave of the device at this off-band-edge frequency.

S12. Wavefront-shaping vs wavefront-selective regimes

A plane wave at normal incidence excites a resonant frequency depending on the local value of the phase gradient [as in Eqn. (92)]:

$$\omega_r(x) = \omega_0 + \frac{b}{2}\left(\frac{\partial \Phi}{\partial x}\right)^2 . \tag{112}$$

If the range of frequencies as a function of position is larger than the linewidth of the resonance, the reflectance will naturally be low, meaning we have a wavefront-selective device. In a metalens, the gradient varies between a value of 0 (at the center of the device) to a value $k_0\mathrm{NA}$. Hence the range of resonant frequencies is $\Delta\omega_r = \frac{|b|}{2}\left(k_0\mathrm{NA}\right)^2$. Enforcing $\Delta\omega_r < d\omega$, where $d\omega = \omega_0 / Q$ is the linewidth of the resonance, we arrive at the condition for the wavefront-shaping regime:

$$\mathrm{NA} < \sqrt{\frac{2/k_0}{|b|Q}}. \tag{113}$$

Notably, the nonlocality length of the band-edge mode is $\xi_0 = \mathrm{Re}[\xi(\omega_0)] = \sqrt{b\tau_r} = \sqrt{bQ/\omega_0}$, which is the characteristic distance that the q-BIC travels in-plane before coupling out. We may rewrite Eqn. (113) in terms of the nonlocality length as

$$\mathrm{NA} < \frac{1}{\sqrt{2}\pi}\frac{\lambda_0}{\xi_0}. \tag{114}$$

S13. Spatial selectivity and eigen-waves

Here, we comment on the expanded understanding of the eigen-wave in these systems. The rigorous definition provided here makes precise the underlying physics demonstrated in Ref. [2] and allowed extension of the concept to frequencies off the band edge and to eigen-waves with submaximal reflectance. We highlight two fundamental features gleaned in the resulting study: (i) there is a strong relationship between the eigen-waves and the point response of the system and (ii) the eigen-waves (regardless of frequency) are best understood as the superposition of two reciprocal reflection events. Regarding (i), the

eigen-wave is not only the wave that, when incident, reflects with maximal efficiency to its time-reversed copy, it is also highly characteristic of the spatial selectivity of the device. We may understand this further by considering eigenvectors other than the one with highest reflectance (i.e., what we call the 'principle' eigen-wave). As shown in Fig. S8, which depicts the eigenvalues for a nonlocal metalens at the band-edge frequency (sorted in descending order of magnitude), the reflectance of the eigenvectors drops quickly away from the principle eigen-wave. Considering the eigendecomposition of the matrix (i.e., $\mathrm{P}^{*}\mathrm{P} = U\Lambda U^{-1}$, where U is the matrix composed of the eigenvectors and Λ is the diagonal matrix whose diagonal contain the corresponding eigenvalues), we may also consider the reflectance of an arbitrary wave by the appropriate sum of the inner products of the eigenvectors with the incident wave, weighted by the appropriate eigenvalues. With this perspective and in light of Fig. S8, it is immediately clear why the eigen-wave characterizes the spatial selectivity: only incident waves with large inner products (i.e., high degree of match) with the principle eigen-wave have substantial reflectance.

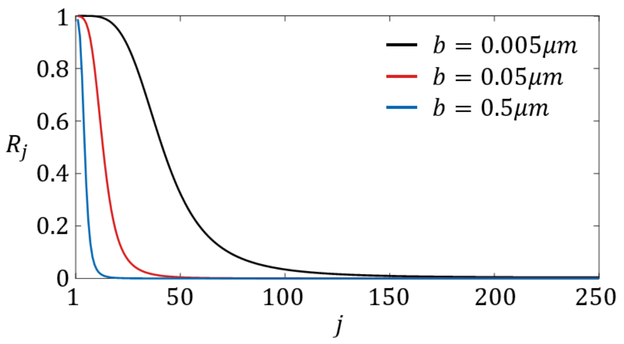

Figure S8. Sorted eigenvalues R_j for a nonlocal metalens with three different values of b, showing increased selectivity (i.e., an effectively smaller basis) to incoming waves as b increases.

S14. Thermal metasurfaces off the band-edge frequency

Here, we apply STCMT to study thermal metalenses as a function of frequency. Figure S9 depicts the computed response for three metalenses with varying Q-factors, $Q=10^2$, $Q=10^3$, and $Q=10^4$. Figures S9(a,c,e) show the response in the space-frequency domain, showing the response roughly follow a parabolic condition ($b=0.15\mu m$ here) while demonstrating increasing selectivity to the position of a point source as the Q-factor increases, especially off the band-edge. Also shown is the selectivity to the spin of the point source. Figures S9(b,d,f) show spatial absorption maps at example wavelengths of interest, showing the splitting of the focal spot into two, as is Fig. S7. Interestingly, as the Q-factor grows, the off-band-edge absorption diminishes rapidly. This implies having a high Q-factor not only decreases the FWHM at the band-edge frequency, but also limits the range of frequencies away from the band-edge contributing meaningful absorption.

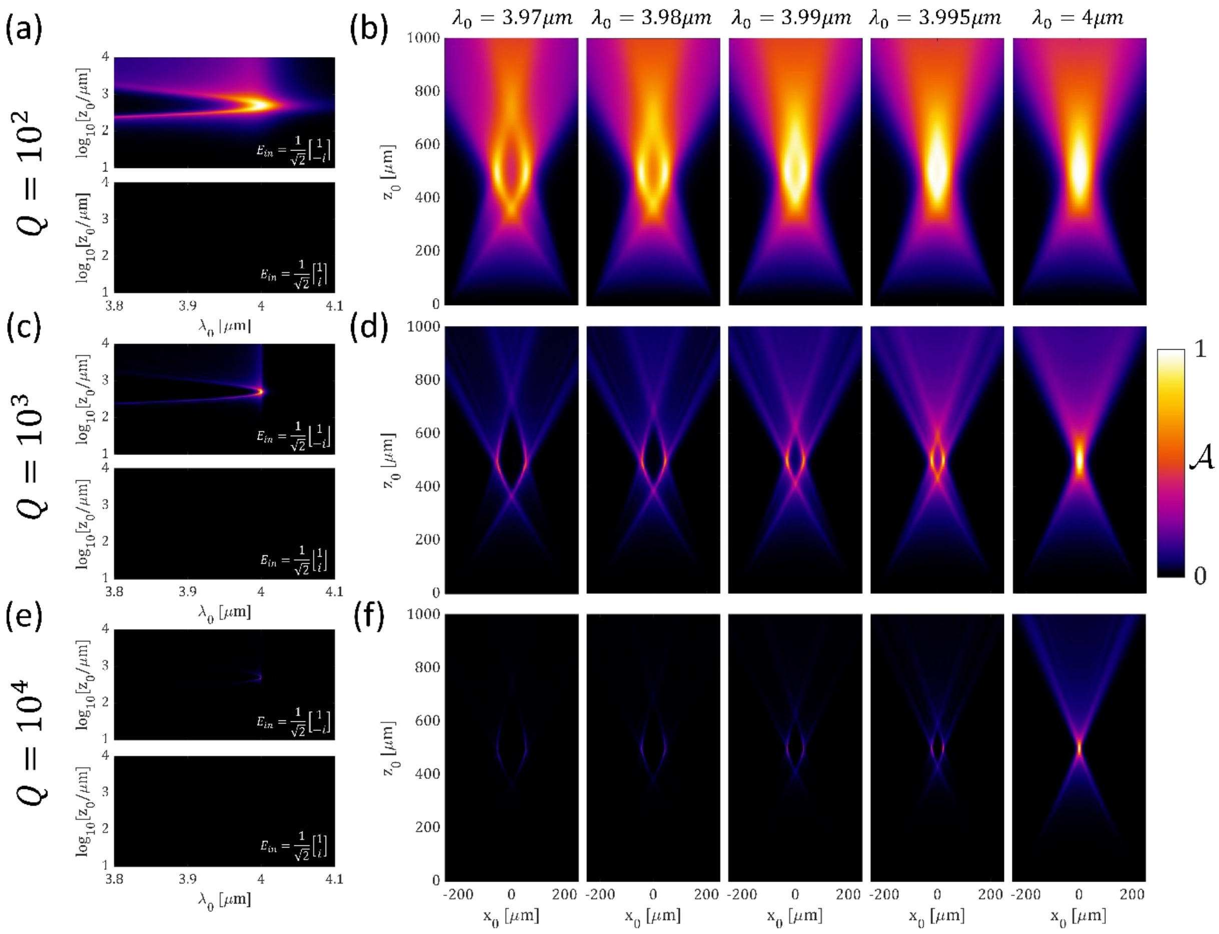

Figure S9. Thermal metalenses as a function of frequency. (a,c,e) Absorption as a function free-space wavelength λ_0 and position of a point source placed at $(0, z_0)$ for the two spin states. (b,d,f) Absorption for the selected spin state as a function of point sources placed at (x_0, z_0) for select wavelengths.

S15. Applicability to local and nonlocal metasurface design

We identify a few key lessons applicable to the broader field of metasurfaces as follows. (i) The nonlocal kernels contain all the information of the functionality of the device, described in the space-frequency domain; (ii) the scattering matrix defined by input and output momenta contains this same information, related to the nonlocal kernels by a mixed Fourier transform; (iii) the nonlocal kernels of an aperiodic device may be approximately constructed by reference to a library of nonlocal kernels of individual structures; and (iv)

the scattering matrix of the composite device is then retrievable by a suitable mixed Fourier transformation.

Additionally, we identify the lessons particular to aperiodic nonlocal metasurfaces as: (i) the nonlocal phase gradient requires vertical asymmetry in the case of unity efficiency, imparting equal and opposite momentum from above or below; (ii) the eigen-waves of the device are composed of a superposition of two waves that reflect into each upon interaction with a nonlocal metasurface; (iii) the eigen-waves closely characterize the point response function of the device, and thereby the spatial selectivity; (iv) the degree of nonlocality increases with lifetime and decreases as the band becomes flatter; (v) the spatial selectivity depends on the degree of nonlocality and the range of momenta encoded into the device; and (vi) a nonlocal metasurface may be classified as either wavefront-shaping or wavefront-selective.

S16. Limitations of the present study

Here, we discuss some limitations in these initial studies. First, we note that our analytical study, and indeed all the devices in Refs. [2], was limited to when the local reflection coefficient is near 0. This choice follows the finding in Refs. [3],[4] that this condition yields complete control over the q-BIC scattering phase. Future work is needed to go beyond this condition and will be aided by the insights of the STCMT. Second, we note that the parabolic approximation of the band structure fails when the spatial frequencies become too high, implying either more terms are necessary in the Taylor expansion and likely requiring purely numerical approaches. Third, we assumed throughout that the

eigenpolarization is independent of incident angle; yet studies on BICs have demonstrated polarization vortices in the momentum-frequency domain [8]. These systems may be captured by modeling the polarization dependence and including its variance in the Fourier transforms. Fourth, we assumed that the local Fresnel coefficient was independent of incident angle (i.e., the reflection coefficient is 0 for all spatial frequencies); yet it is well known that all interfaces have unity reflectance at grazing incidence. The consequence of this assumption is the absence of highly localizes features such as those seen in Fig. S1 for a bare interface, and this simplification yielded the convenient and insightful analytical relations we found. However, if the nonlocality length is comparable or smaller than the characteristic extent of these features from the local scattering, and if we operate near grazing incidence, our approximation is invalid, and we must return to numerical methods. Fifth, while the extension to 2D nonlocal metasurfaces is straightforward in the case of isotropic dispersion (briefly overviewed in Supplementary Section S10), more expansion terms must be included in Eqn. (5) of the main text to account for dispersion that varies as a function of azimuthal direction. Finally, we remind the reader of the spurious numerical results born of sparse discretization, implying that the bandwidth of the STCMT is not unlimited and increasing it requires increasing the resolution and therefore size of the nonlocal matrices.

References

[1] H. A. Haus, Waves and Fields in Optoelectronics, New Jersey, Englewood Cliffs: Prentice-Hall, (1984).

[2] S. Fan, W. Suh, and J. D. Joannopoulos, “Temporal coupled mode theory for Fano resonances in optical resonators,” *J. Opt. Soc. Am. A*, **20**, 569–573, (2003).

[3] A.C. Overvig, N. Yu, and A. Alù, “Chiral Quasi-Bound States in the Continuum”, *Phys. Rev. Lett.* **126**, 073001 (2021).

[4] A.C. Overvig, Y. Kasahara, G. Xu, and A. Alù, “Observation of a polarization-agnostic geometric phase in nonlocal metasurfaces”, *arXiv:2302.13215* (2023).

[5] A.C. Overvig and A. Alù, “Wavefront-Selective Fano Resonant Metasurfaces”, *Adv. Photonics* **3**, 026002 (2021).

[6] H. Zhang, O.D. Miller, “Quasinormal Coupled Mode Theory”, arXiv:2010.08650 (2020).

[7] X. Buet, E. Daran, D. Belharet, F. Lozes-Dupuy, A. Monmayrant, and O. Gauthier-Lafaye, “High angular tolerance and reflectivity with narrow bandwidth cavity0resonator-integrated guided-mode resonance filter”, *Optics Express* **20**, 9322- (2012).

[8] H.M. doeleman, F. Monticone, W. den Hollander, A. Alù, and A.F. Koenderink, “Experimental observation of a polarization vortex at an optical bound state in the continuum”, Nature Photonics 12, 397-401 (2018).

S17. Example MATLAB code, reproducing Fig. 5b of the maintext

Initialize problem and device variables

```
close all; clearvars;

lams = linspace(1.55,1.65,1001); %sampled wavelength
ws = 2*pi./lams; %sampled frequencies, units c=1

%resonance characteristics
lam0 = 1.6104; %resonant wavelength, microns
w0 = 2*pi./lam0; %resonant frequency
k0 = 2*pi/lam0; %resonant momentum
taur = 250; %lifetime
b = 0.021; %band curvature

%Device properties
n = 1.45; %refractice index of surrounding medium (glass)
a= .4; %pitch of meta-units, in microns
W0 = 75; %width of device, in microns
NA = .4; %numerical aperture
f0 = W0/2*sqrt(1/NA^2-1); %focal length
```

Construct and visualize example reflection scattering kernel

```
x = 0:a:(W0-a); %meta-unit positions
x = x-mean(x);
xp = x; %primed coordinates
[Xp,X] = meshgrid(xp,x);
dxxp = abs(X-Xp); %position difference matrix, pre-computed

dw=0; %shift from resonant frequency, w0, for visualization
xi = sqrt(1i*b*taur/2./(1-1i*(dw)*taur)); %nonlocality length

%phase profile in x, xp:
PHI = exp(-1i*k0*(sqrt(X.^2+f0^2)-f0)*n).*exp(-1i*k0*(sqrt(Xp.^2+f0^2)-f0)*n);

%complex reflection kernel:
RHO = a.*xi.*exp(-dxxp./xi)/(b*taur).*PHI;

%map amplitude/phase to hsv
phase = (angle(RHO)+pi)/(2*pi);
phi0 = 0; %reference phase
phase = mod(phase-phi0,1);
sze = size(RHO);
IM = ones([sze(1),sze(2),3]);
IM(:,:,1) = phase;
IM(:,:,2) = (abs(RHO)/max(abs(RHO(:)))).^2;
IM(:,:,3) = 1-0.5*(abs(RHO)/max(abs(RHO(:)))).^2;
IM = hsv2rgb(IM);
fh = figure('position',[0,0,600,600]);
ah = axes('Units','Normalize','Position',[0 0 1 1]);
imagesc(x,x,IM);
xlabel('x [\mum]');
ylabel('y [\mum]');
```

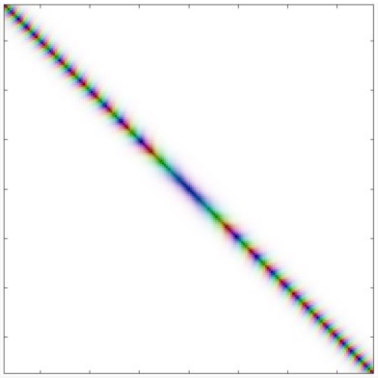

Sweep over position z0 and wavelength lams

```
z0s = 10.^linspace(1,5,101); %sampled source positions
Rs = zeros(length(lams),length(z0s)); %storage variable for reflectance

for j=1:length(lams)
    %update xi and RHO for this wavelength
    xi = sqrt(1i*b*taur/2./(1-1i*(ws(j)-w0)*taur));
    RHO = a.*xi.*exp(-dxxp./xi)/(b*taur).*PHI;

    for k=1:length(z0s)
        %construct incident wavefront corresponding to point source at z0
        Ein = exp(1i*w0*sqrt((xp).^2+z0s(k)^2)*n); %incident field
        Pin = norm(Ein).^2; %incident power, for normalization

        Eout = RHO*Ein.'; %outgoing field computed from scattering equation
        Pout = norm(Eout).^2; %output power
        Rs(j,k) = Pout/Pin; %total reflectance
    end
end
```

Visualize map of reflectance

```
asp = [2,1,1];
figure;
imagesc(lams,log10(z0s),Rs.');
ylabel('log_{10}(z_0/\mum)');
xlabel('\lambda_0 [\mum]');
set(gca,'YDir','normal')
colormap gray
pbaspect(asp);
cbh = colorbar;
maxR = max(Rs(:));
clim([0,1]);
cbh.YTick=[0,1];
set(gca,'YTick',[1,2,3,4,5]);
set(gca,'XTick',[1.55,1.6,1.65]);
set(gca,'fontsize',16);
set(gca,'fontname','cambria math')
```

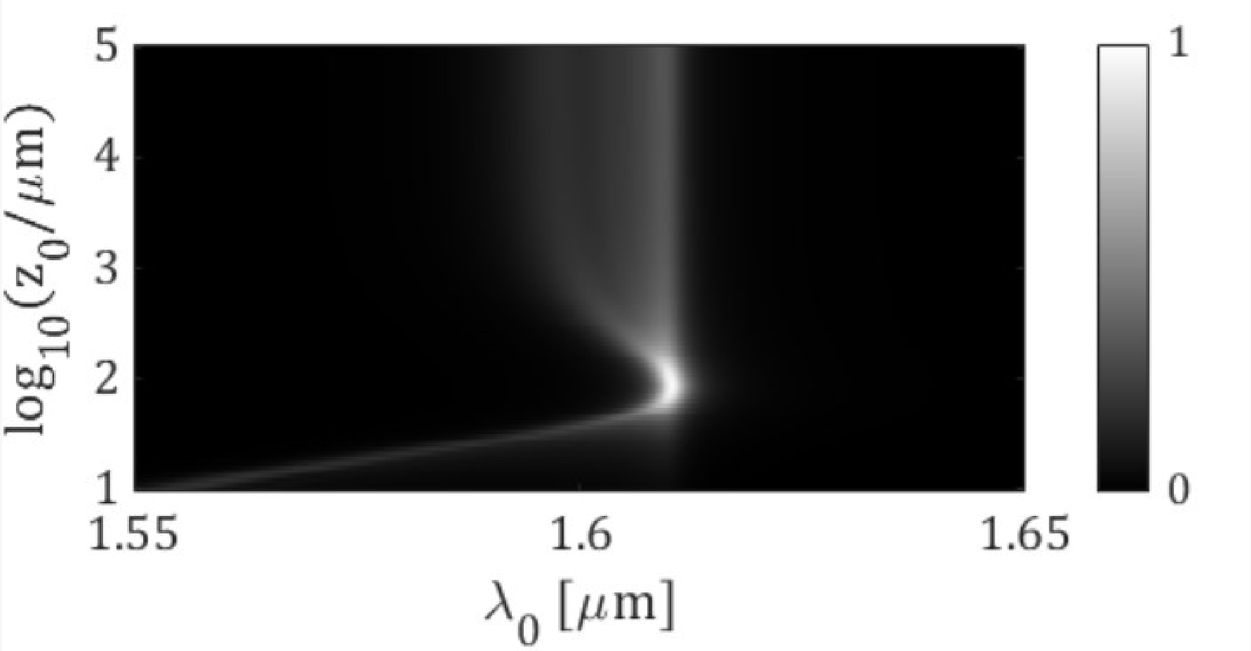

S18. Example MATLAB code, reproducing thermal metalens from Fig. 6

Initialize problem and device variables

```
close all; clearvars;

lam = 4; %sampled wavelength (here, resonant wavelength)
f = 1./lam; %sampled frequency, units c=1

%choose polarization basis [e,ec], here CP
e = [1,-1i]; e = e/norm(e);
ec = [conj(e(2)),-conj(e(1))]; ec = ec/norm(ec);

%resonance characteristics
lam0 = 4;  %resonant wavelength, microns
f0 =1./lam0; %resonant frequency, c=1
b = .15; %band curvature
Q = 10000; %Q-factor
taur = Q*pi*lam0; %radiative lifetime
gr = 1/taur; %radiative scattering rate
gnr = gr; %non-radiative scattering rate
taut = 1./(gr+gnr); %total lifetime

%Device properties
W = 500; %width of device, in microns
fd = 500; %focal distance, in microns
a = 1.5; %pitch of meta-units, in microns
```

Construct and visualize example reflection scattering kernel

```
x = 0:a:W; %meta-unit positions
x = x-mean(x);
xp = x; %primed coordinates
[Xp,X] = meshgrid(xp,x);
dxxp = abs(X-Xp); %position difference matrix, pre-computed

%spatial profile of theta and phi
theta = -(2*pi*f0)*sqrt(xp.^2+fd^2); %angle of local birefringent element
phi = theta-pi/4; %scattered linear polarization state by nonlocal element
[Thetap,Theta] = meshgrid(theta,theta);
[Phip,Phi] = meshgrid(phi,phi);

%scattering vector |d>=[d1,d2]
d1 = 1i*sqrt(gr)*(cos(phi)-1i*cos(2*theta-phi));
d2 = 1i*sqrt(gr)*(sin(phi)-1i*sin(2*theta-phi));

%Compute elements of the outer product D=|d><d*|, pointwise
[D1p,D1] = meshgrid(d1,d1);
[D2p,D2] = meshgrid(d2,d2);
D11 = D1p.*D1;
D12 = D2p.*D1;
D21 = D1p.*D2;
D22 = D2p.*D2;

%local scattering matrix
C11 = diag(cos(2*theta));
C12 = diag(sin(2*theta));
C21 = diag(sin(2*theta));
C22 = diag(-cos(2*theta));
C = [C11, C12; C21, C22];

%compute nonlocality length
xi = sqrt(1i*b*taut/2./(1-1i*(f-f0)*taut));

%compute reflection kernel matrix
% RHO0 = - 2*a.*xi.*exp(-dxxp./xi)/(b);
RHO0 =  a.*xi.*exp(-dxxp./xi)/(b);
RHO11 = RHO0.*D11;
RHO12 = RHO0.*D12;
RHO21 = RHO0.*D21;
RHO22 = RHO0.*D22;
RHO = [RHO11, RHO12; RHO21, RHO22];

%scattering matrix
S = C + RHO;

figure;
imagesc(xp,x,abs(C));
axis equal tight
axis off
colormap gray;
cbh = colorbar;
cbh.YTick = [0,1];
clim([0,1]);

figure;
imagesc(xp,x,abs(RHO));
axis equal tight
axis off
colormap gray;
cbh = colorbar;
cbh.YTick = [0,max(abs(RHO(:)))];
```

```
figure;
imagesc(xp,x,angle(RHO));
axis equal tight
axis off
colormap hsv
cbh = colorbar;
cbh.YTick = [-pi,0,pi];
clim([-pi,pi]);
```

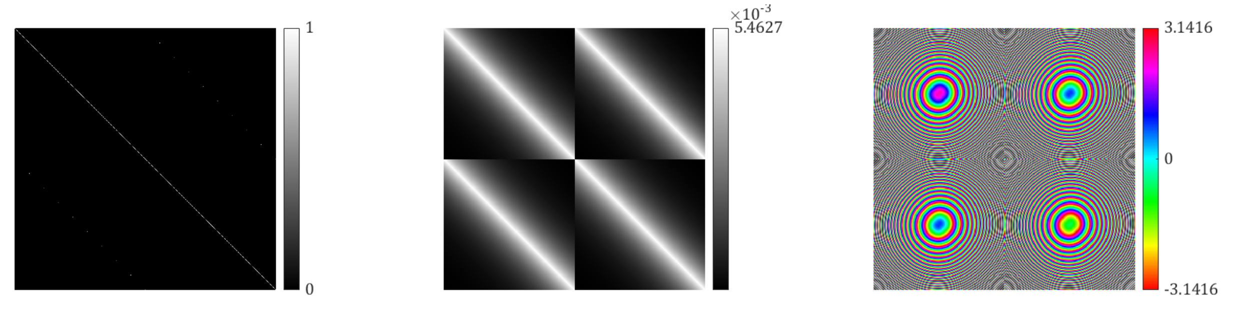

Sweep over positions x0 and z0 of point source

```
x0s = linspace(-W/2,W/2,201); %sampled x positions of point source
z0s = linspace(0,2*fd,201); %sampled z positions of point source

Rs = zeros(length(x0s),length(z0s)); %storage variable for first polarization
Rsc = zeros(length(x0s),length(z0s)); %storage variable for second polarization

for j = 1:length(x0s)
    for k=1:length(z0s)
        %construct incident wavefront of polarized point source at (x0,z0)
        Ein0 = exp(1i*2*pi*f0*sqrt((xp-x0s(j)).^2+z0s(k)^2));
        Ein = [e(1)*Ein0, e(2)*Ein0]; %input field
        Pin = norm(Ein).^2; %input power

        Eout = S*Ein.'; %reflected field
        Pout = norm(Eout).^2; %reflected power
        Rs(j,k) = Pout/Pin; %reflectance

        %same but for second polarizaton
        Ein = [ec(1)*Ein0, ec(2)*Ein0];
        Pin = norm(Ein).^2;

        Eout = S*Ein.';
        Pout = norm(Eout).^2;
        Rsc(j,k) = Pout/Pin;
    end
end
```

Visualize map of reflectance

```
figure;

imagesc(x0s,z0s,1-Rs.');
```

```
set(gca,'YDir','normal');
% pbaspect([2,1,1]);
colormap hot;
set(gca,'FontName','Cambria Math');
set(gca,'Fontsize',16);
xlabel('x_0 [\mum]');
ylabel('z_0 [\mum]');
axis equal tight
cbh = colorbar;
cbh.YTick = [0,1];
clim([0,1]);

figure;
imagesc(x0s,z0s,1-Rsc.');
set(gca,'YDir','normal');
% pbaspect([2,1,1]);
colormap hot
set(gca,'FontName','Cambria Math');
set(gca,'Fontsize',16);
xlabel('x_0 [\mum]');
ylabel('z_0 [\mum]');
axis equal tight
cbh = colorbar;
cbh.YTick = [0,1];
clim([0,1]);
```

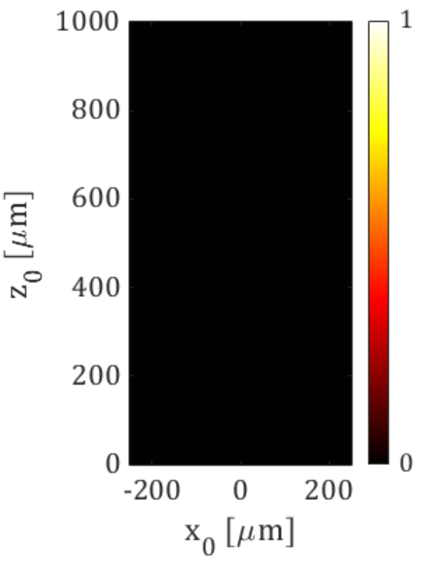

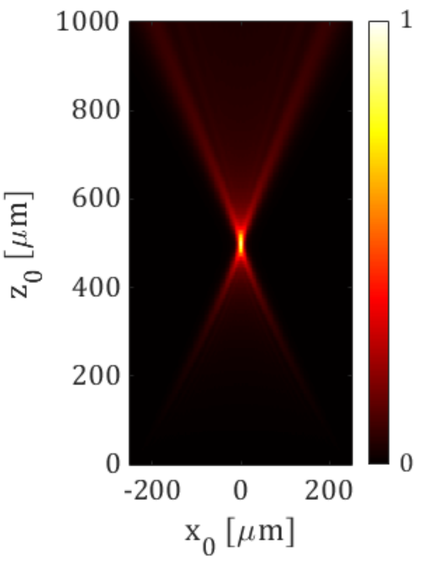

Supplement: Supplementary file 1 — Supplementary Materials [file 41377_2023_1350_MOESM1_ESM.pdf]
